# Thermoelectric plastics: from design to synthesis, processing and structure–property relationships

**DOI:** 10.1039/c6cs00149a

**Published:** 2016-07-07

**Authors:** Renee Kroon, Desalegn Alemu Mengistie, David Kiefer, Jonna Hynynen, Jason D. Ryan, Liyang Yu, Christian Müller

**Affiliations:** a Department of Chemistry and Chemical Engineering , Chalmers University of Technology , 41296 Göteborg , Sweden . Email: christian.muller@chalmers.se

## Abstract

Thermoelectric plastics are a class of polymer-based materials that combine the ability to directly convert heat to electricity, and *vice versa*, with ease of processing. Potential applications include waste heat recovery, spot cooling and miniature power sources for autonomous electronics. Recent progress has led to surging interest in organic thermoelectrics. This tutorial review discusses the current trends in the field with regard to the four main building blocks of thermoelectric plastics: (1) organic semiconductors and in particular conjugated polymers, (2) dopants and counterions, (3) insulating polymers, and (4) conductive fillers. The design and synthesis of conjugated polymers that promise to show good thermoelectric properties are explored, followed by an overview of relevant structure–property relationships. Doping of conjugated polymers is discussed and its interplay with processing as well as structure formation is elucidated. The use of insulating polymers as binders or matrices is proposed, which permit the adjustment of the rheological and mechanical properties of a thermoelectric plastic. Then, nanocomposites of conductive fillers such as carbon nanotubes, graphene and inorganic nanowires in a polymer matrix are introduced. A case study examines poly(3,4-ethylenedioxythiophene) (PEDOT) based materials, which up to now have shown the most promising thermoelectric performance. Finally, a discussion of the advantages provided by bulk architectures *e.g.* for wearable applications highlights the unique advantages that thermoelectric plastics promise to offer.

## Abstract 2

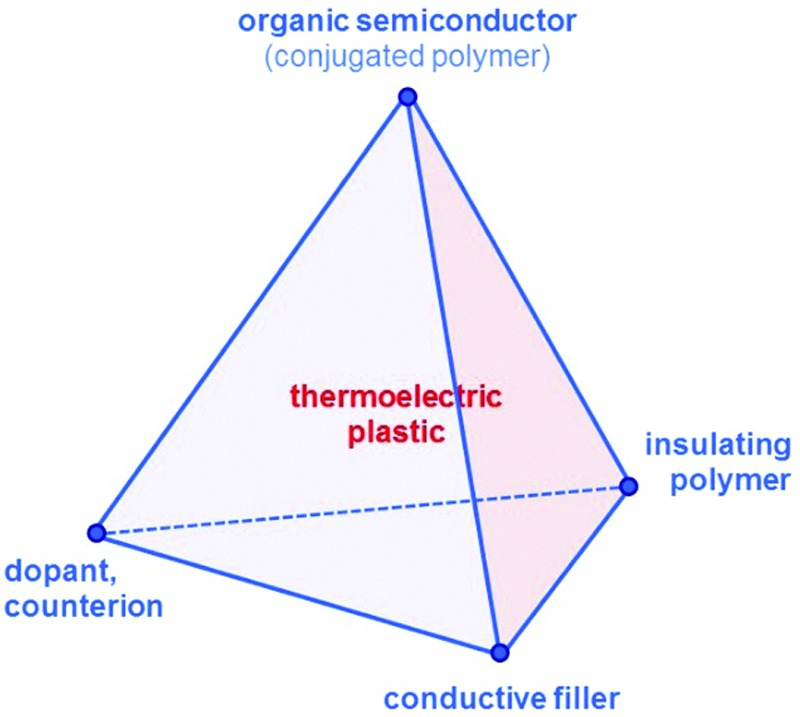
Thermoelectric plastics are a class of polymer-based materials that combine the ability to directly convert heat to electricity, and *vice versa*, with ease of processing.

Key learning points

(1) Thermoelectric plastics combine the ability to directly convert heat to electricity, and *vice versa*, with ease of processing.

(2) Organic semiconductors such as conjugated polymers and fillers such as carbon nanotubes, graphene and inorganic nanowires can be used as the primary charge-conducting materials.

(3) Doping permits the optimisation of thermoelectric properties, but affects processability and nanostructure of the semiconductor.

(4) Insulating binders and matrix polymers can be added to adjust the rheological and mechanical properties.

(5) Plastic processing enables the bulk architectures that are needed for optimally designed thermoelectric devices.

## 1. Introduction

Heat is an abundant but often wasted source of energy. If an electrical conductor or semiconductor is put in contact with such a heat source an electrical potential is generated, which can be used to drive an electric current. Thermoelectric generators exploit this so-called Seebeck effect, discovered in 1821 by Thomas J. Seebeck, to directly convert heat into electricity. Conversely, when an electrical current is applied to the same type of device, the analogous Peltier effect, discovered in 1834 by Jean Peltier, can be used to convert electrical energy to thermal energy, which is of interest for spot cooling or heating applications. Thermoelectric generators are solid-state devices that contain no moving parts and thus can operate over a long period of time without significant maintenance. Small heat sources and limited temperature differences are sufficient to drive thermoelectric generators, which makes these devices interesting for applications where traditional dynamic heat engines cannot be employed.

At present the high cost of commercially available thermoelectric technologies prevents their more widespread usage. The current state-of-the-art inorganic materials are metal alloys such as bismuth telluride, which are expensive to micro-fabricate and contain moderately to highly toxic elements such as tellurium, antimony and lead. Therefore, inorganic thermoelectric devices are mostly limited to niche applications such as unaffordable wrist watches that harvest body heat and radioisotope thermoelectric generators that power spacecraft and arctic lighthouses by converting the heat released from a decaying radioactive material, as well as Peltier elements that cool scientific and high-end electronic equipment.

In contrast, thermoelectric plastics, *i.e.* malleable polymer-based thermoelectric materials, that can be readily shaped into a wide range of thin-film to bulk architectures offer an interesting alternative since they promise more cost-effective processing from melt or solution. In particular, conjugated polymers are of interest. They are based on abundant elements – mainly carbon, oxygen, nitrogen and sulphur – and their precise chemical structure can be easily tuned by exploiting the vast toolbox of polymer chemistry. Additional advantages are their mechanical robustness and flexibility, low weight and typically low thermal conductivities.

The know-how regarding high throughput coating and printing of large-area electronics such as polymer solar cells, logic circuits and flexible displays has advanced significantly over the last couple of years and can be readily transferred to the fabrication of thermoelectric modules, as demonstrated in a proof-of-concept study by Søndergaard *et al.* ^1^ Textile manufacturing and 3D printing, which have only received limited attention for thin-film organic electronics, offer interesting alternatives since they are readily compatible with the millimetre-thick dimensions of an optimally designed thermoelectric generator.

There is a broad range of potential application areas for thermoelectric plastics. Large-area devices are promising for waste heat recovery on an industrial scale for temperatures up to about 200 °C, *e.g.* by cladding chimneys, pipes or high-voltage power cables, and may provide cooling in the case of, for instance, car seats or data centres. Instead, flexible, small-scale thermoelectric generators have the potential to expand the reach of autonomous electronics that will make up tomorrow's *Internet of Things*, possibly powered by body heat in the case of e-textiles.

### 1.1. Thermoelectric performance parameters

Any conductor or semiconductor that is exposed to a temperature gradient Δ*T* = *T*_hot_ – *T*_cold_ will experience the Seebeck effect, *i.e.* accumulation of charge carriers, which arises due to diffusion from the hot to the cold end balanced by the resulting internal electric field, and gives rise to a potential difference Δ*V*. For a given material, the temperature-dependent Seebeck coefficient *α*(*T*) = –d*V*/d*T*, which can be considered as the entropy per charge carrier, describes the potential difference that arises per unit temperature difference. For small changes in temperature *α*(*T*) is almost constant and thus we obtain:1
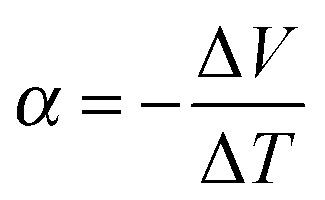
 By convention the sign of *α* is given by the potential of the cold side with respect to the hot side and thus indicates the type of majority charge carriers, *i.e.* electrons or holes, with *α* < 0 for n-type and *α* > 0 for p-type semiconductors.

A material that is suitable for thermoelectric energy harvesting must, besides a high *α*, also feature a high electrical conductivity *σ* and low thermal conductivity *κ*, in order to minimise both electrical losses through Joule heating as well as thermal losses due to equilibration between the hot and cold temperature reservoir. These requirements can be conveniently summarised by the dimensionless figure of merit *ZT*:2
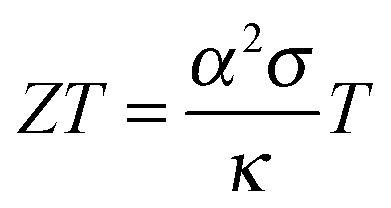
where *T* is the absolute temperature. The inorganic benchmark material bismuth telluride offers a *ZT* ∼ 1 at room temperature, whereas some p-type organic materials now reach values of *ZT* ≥ 0.1. Since, in particular, the thermal conductivity can be challenging to measure, the power factor *α* ^2^ *σ* is often used instead of *ZT* for the purpose of comparing the thermoelectric performance of different materials.

The thermoelectric parameters *α*, *σ* and *κ* are intimately linked and can vary with temperature (*cf.*eqn (1)), which complicates optimisation and typically requires a compromise. For example, the electrical conductivity is described by the product of charge carrier concentration *n* and charge carrier mobility *μ*:

3 *σ* = *qnμ*

where *q* is the electric charge of a charge carrier. Accordingly, *σ* strongly depends on the nanostructure of a semiconductor, which affects *μ*, and increases with *n* upon doping, *i.e.* the process of adding charge carriers to a semiconducting material. In highly doped organic semiconductors *σ* reaches values of 10^1^ to more than 10^3^ S cm^–1^. On the other hand, the Seebeck coefficient, which is related to the density of states, *i.e.* the energetic landscape of a material, tends to decrease upon doping. Typical values for *α* range from 10^3^ μV K^–1^ for undoped (intrinsic) semiconductors (*e.g.*, undoped conjugated polymers) to 10^2^ to 10^1^ μV K^–1^ for moderately to heavily doped (extrinsic) semiconductors, and *α* < 10^1^ μV K^–1^ for good conductors, including metals. Although the density of states of many pristine organic semiconductors has been studied in detail, there is only limited knowledge regarding the effect of molecular doping as it also strongly impacts the nanostructure.^2^ We refer the reader to an excellent review by Bubnova and Crispin that elucidates the solid-state physics of conjugated polymers.^3^

To estimate the figure of merit a careful determination of the thermal conductivity is required, which contains contributions from both phonons and electrons:

4 *κ* = *κ*_phonon_ + *κ*_electronic_

According to the Wiedemann–Franz law, the electronic part is related to the electrical conductivity by the so-called Lorentz number *L*:

5 *κ*_electronic_ = *LσT*

The validity is generally established for inorganic semiconductors, where *L* adopts the close-to-constant Sommerfeld value of 2.44 × 10^–8^ W Ω K^–2^, and appears to be applicable to at least some organic materials (*cf.* Section 7). Materials with a low electrical conductivity of, *e.g.*, 1 S cm^–1^ will feature a negligible electronic contribution to the thermal conductivity *κ*_electronic_ < 0.001 W m^–1^ K^–1^. The measured thermal conductivity is then equal to the phonon contribution, which for many isotropic insulating plastics has a value of *κ*_phonon_ ∼ 0.1 to 0.5 W m^–1^ K^–1^. Instead, for values of *σ* ∼ 100 S cm^–1^ and above *κ*_electronic_ is likely to approach or even exceed *κ*_phonon_ and thus must be taken into account.

### 1.2. Basic design of a thermoelectric generator

**Fig. 1:**
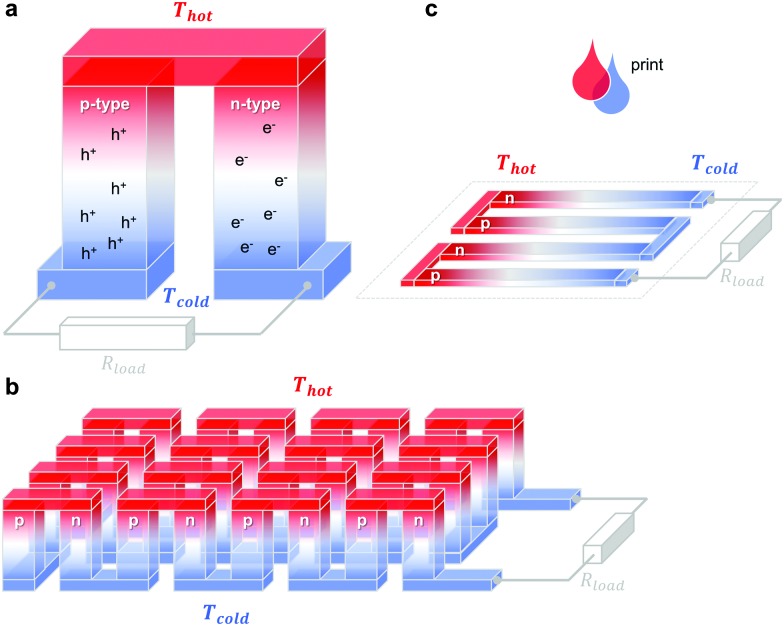
Schematics of (a) a thermoelectric element, which comprises one n- and one p-type thermoelectric leg that experience a temperature gradient Δ*T* = *T*_hot_ – *T*_cold_ leading to charge accumulation at the cold ends, (b) a conventional thermoelectric module that comprises an array of elements, which are connected electrically in series but thermally in parallel, and (c) an in-plane (printable) array of elements. A load resistance *R*_load_ is connected to close the circuit, resulting in maximum power generation when *R*_load_ = *R*_int_ but maximum power conversion efficiency when 
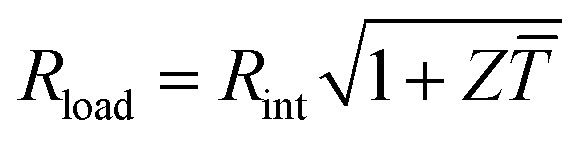
 (*cf.*eqn (7) and (8)).

A single piece of thermoelectric material only produces an insufficiently low thermovoltage of typically not more than a few millivolts when exposed to a large temperature gradient, which cannot be used to power electronic components (a typical requirement is one volt). Hence, it is necessary to connect an array of so-called legs that are connected thermally in parallel but electrically in series. Each pair of legs forms a thermoelectric element, *i.e.* a thermocouple, and represents the smallest repeat unit of a thermoelectric generator (Fig. 1). There is an optimal leg length (and area coverage) of typically several 100 micrometres to millimetres that provides a balance between electrical and thermal losses, which are minimised in the case of short and long legs, respectively.

The two legs of each element must be composed of dissimilar materials with different Seebeck coefficients *α*_1_ and *α*_2_, so that the resulting thermovoltages do not exactly cancel, *i.e. α*_1_ – *α*_2_ ≠ 0. The most efficient design, which is commonly chosen for inorganic materials, connects n- and p-type legs so that the thermovoltage of each leg contributes to the total open-circuit voltage of the generator:

6 *V*_oc_ = *N*·(*α*_1_ – *α*_2_)·Δ*T*

where *N* is the number of elements (note that *α*_1_ and *α*_2_ are of opposite sign).

The maximum power *P*_max_ is obtained when the external load and the internal resistance of the generator match, *i.e. R*_load_ = *R*_int_ (*cf.* Fig. 1), and is related to *V*_oc_ according to:

7 *P*_max_ = *V*_oc_^2^/4*R*_int_

Here, it is interesting to note that for a given area coverage, *V*_oc_ increases with *N* whereas *P*_max_ is independent of the number of elements since *R*_int_ increases accordingly.

The power conversion efficiency *η* = *P*/*Q* with which a thermoelectric leg turns heat *Q* into useful power *P* increases with its figure of merit, and is maximised when 
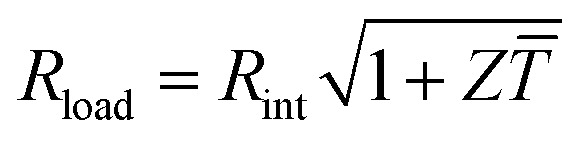
, which implies that a generator can operate in either maximum power or power conversion efficiency mode. The maximum efficiency is then given by:8
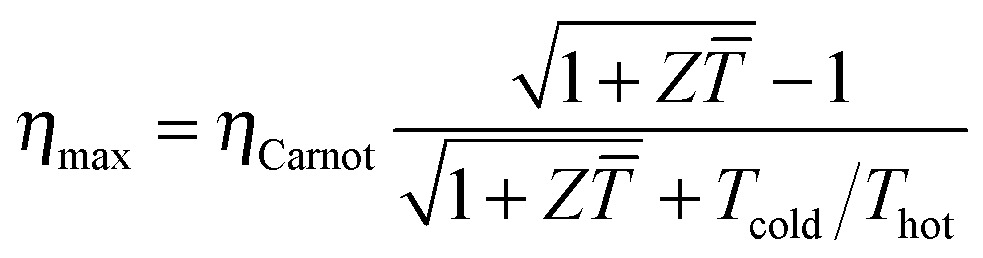
where *T* = (*T*_hot_ + *T*_cold_)/2 (the same relationship is obtained for a generator if its n- and p-type legs have the same thermoelectric properties). Just as all other heat engines, the efficiency is limited by the Carnot efficiency *η*_Carnot_ = Δ*T*/*T*_hot_. The second term represents the internal losses that arise due to Joule heating and heat conduction.

As environmentally stable n-type doping of organics is more challenging to realise,^4^ it is worth considering alternative designs where each thermoelectric element comprises one p-type semiconductor leg together with a regular conductor that has a very low Seebeck coefficient (*e.g.*, *α* ∼ 1.5 μV K^–1^ for silver). Several studies have explored such designs where essentially only the p-type semiconductor leg contributes to the open-circuit voltage of the generator.^1,5,6^ According to eqn (6) and (7) replacing n-type legs with a regular conductor reduces *P*_max_ by a factor of about two, assuming that both *V*_oc_ and *R*_int_ are halved.

### 1.3. Overview of organic thermoelectrics

The discovery that iodine-doped polyacetylene can display a high electrical conductivity,^7^ for which the 2000 Nobel Prize in Chemistry was awarded, sparked an early interest in organic thermoelectrics. The best results were achieved with iodine-doped, stretch-aligned polyacetylene,^8^ which is highly conductive leading to a very high power factor of up to 1350 μW m^–1^ K^–2^ (Table 1) but suffers from poor environmental stability. Other materials such as camphorsulfonic acid (CSA) doped, stretch-aligned polyaniline, which offers very good environmental stability, were found to display much lower power factors of up to 5 μW m^–1^ K^–2^.^9^ Following these early advances organic thermoelectrics only received modest interest until 2008 because of the lack of doped materials that are both environmentally stable and offer higher power factors. Instead, the organic electronics community focused on intrinsic semiconductors as active materials *e.g.* for light-emitting diodes (LEDs), field-effect transistors (FETs) and photovoltaics.

**Table 1:** Record thermoelectric performance among the materials discussed in this review

	Semiconductor	Dopant or counterion	Filler & binder	*σ* (S cm^–1^)	*α* (μV K^–1^)	*α* ^2^ *σ* (μW m^–1^ K^–2^)	*κ* (W m^–1^ K^–1^)	*ZT* at r.t.	Ref.
p-Type	polyacetylene^*a*^	I_2_		60 000	15	1350			8
	PBTTT	FTS		1000	33	110			34
	PEDOT	Tos + TDAE		70	215	324	0.37^*b*^	0.25	12
	PEDOT	PSS		880	73	469	0.33^*b*^	0.42	44
	PEDOT	PSS	SWNT + PVA	950	41	160	0.38^*c*^		14
	PEDOT	PSS	Tellurium nanowires	19	163	71	0.22–0.30^*c*^	0.1	13

n-Type	P(NDIOD-T2)	N-DMBI		0.008	–850	0.6			35
	FBDPPV	N-DMBI		6	–200	25			17
	P3HT		Nitrogen-doped MWNT	10	–10	0.1	0.55^*d*^	10^–5^	42

^*a*^Stretch aligned.

^*b*^In-plane *κ*_∥_.

^*c*^Out-of-plane *κ*_⊥_.

^*d*^Bulk *κ*.

The work by Leclerc *et al.* who studied the thermoelectric properties of a series of polycarbazoles,^10^ and Yu *et al.* who characterised composites of poly(vinyl acetate) (PVA) and carbon nanotubes,^11^ stimulated a series of papers in 2010–2011 that revived the interest in organic thermoelectrics by demonstrating a *ZT* ≥ 0.1 for environmentally stable conjugated polymer-based materials (Table 1). Bubnova *et al.* carefully reduced poly(3,4-ethylenedioxythiophene):tosylate (PEDOT:Tos).^12^ For an optimal p-doping level that struck the best compromise between *σ* and *α*, a maximum power factor *α* ^2^ *σ* ∼ 324 μW m^–1^ K^–2^ could be obtained. At the same time, similar power factors were demonstrated by See *et al.* and Yu *et al.* who used an alternative approach based on nanocomposites of the widely studied conjugated polymer:polyanion blend PEDOT:poly(styrene sulfonate) (PEDOT:PSS) with tellurium nanowires and carbon nanotubes that gave rise to values of *α* ^2^ *σ* ∼ 71 μW m^–1^ K^–2^ and 160 μW m^–1^ K^–2^, respectively.^13,14^

**Fig. 2:**
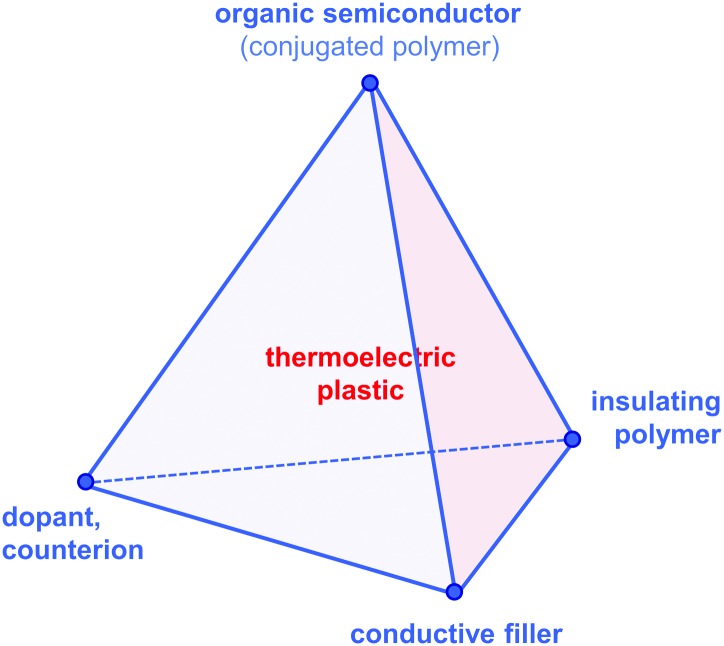
The tetrahedron of thermoelectric plastics, illustrating the four main building blocks: (1) organic semiconductors and in particular conjugated polymers, (2) dopants and counterions, (3) insulating polymers, and (4) conductive fillers such as carbon nanotubes, graphene and inorganic semiconductor nanowires.

Since then, a growing number of papers have been published on the topic of organic thermoelectrics that explore various combinations of up to four building blocks: (1) organic semiconductors and in particular conjugated polymers, (2) dopants and counterions, (3) insulating polymers that can act as matrices or binders, and (4) conductive fillers such as carbon nanotubes, graphene and inorganic nanowires (Fig. 2). Each one of these components serves a specific purpose, which depends on the combination of (1–4). Both the organic semiconductor and filler can be used as the primary charge-conducting components, and dopants permit the modulation of the charge carrier concentration. In the case of carbon nanotubes, graphene and inorganic nanowires, this review will limit the discussion to the use of these materials as fillers in a polymeric matrix, which can be either a conjugated or an insulating polymer. Besides improving the processability of the filler, the use of a polymeric binder is a convenient strategy to adjust the rheological and mechanical properties of thermoelectric plastics.

A very recent development is the exploitation of the dual ability of some organic thermoelectric materials such as PEDOT:PSS to sustain both electric and ionic charge transport. Wang *et al.* have shown that in addition to hole diffusion in the PEDOT phase due to the Seebeck effect, ions in the PSS phase can experience thermophoresis, *i.e.* the so-called Soret effect, and diffuse along a thermal gradient.^15^ Both the Seebeck and Soret effect contribute to the total thermovoltage.

## 2. Chemical design and synthesis of conjugated polymers

Up to now only a few, well-known conjugated polymers have been studied in the context of organic thermoelectrics. However, it can be anticipated that the recent interest in new organic thermoelectric materials will stimulate a surge in the synthesis of specifically designed thermoelectric polymers. Other areas of organic electronics such as organic photovoltaics, where large-area production currently struggles due to its initially efficiency-oriented research, offer valuable insights regarding conjugated polymer design. Aside from efficiency, factors such as cost, processability and stability must be taken into account for the development of a competitive technology. In addition, the whole lifecycle of the material should be considered where a cradle-to-cradle approach with 100% reusability is most desirable. The cost associated with polymer synthesis and processing includes aspects such as (1) the availability of the raw material, (2) scalability of the synthesis, (3) non-toxicity, and (4) melt and/or solution processability (ideally from green solvents such as water or alcohols), which imposes strict design rules on the chemical structure of monomers and polymers. Besides the environmental stability of a material during processing and operation, which is a prerequisite for a long shelf and service life, one should also consider mechanical stability if flexible applications are targeted. Although significant synthetic efforts are required to facilitate a solid fundamental understanding, it is vital that researchers from the start pay attention to the aforementioned applied aspects in order to accelerate the practical use of organic thermoelectrics.

### 2.1. Energy level design of conjugated polymers

**Fig. 3:**
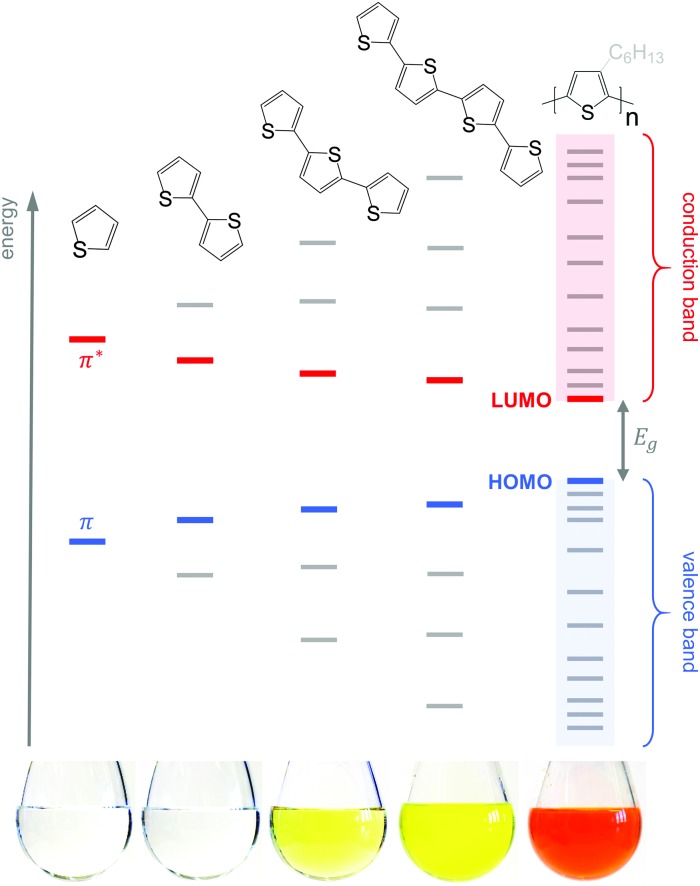
Evolution of the HOMO and LUMO levels as well as bandgap *E*_g_ with increasing number of thiophene repeat units, resulting in valence and conduction bands for polythiophene; the images of 5 g L^–1^ solutions in chloroform illustrate the narrowing of *E*_g_, which leads to a red-shift in absorption (the polymer is P3HT). Note that crystallisation will lead to a further decrease in *E*_g_ due to electron delocalisation across adjacent chain segments.

Conjugated polymers have an alternating single-double bond structure, which gives rise to their semiconducting properties. Chemical coupling of conjugated monomers into long polymer chains leads to orbital interaction and, consequently, to energy level splitting of the π and π* orbitals (Fig. 3). From the π-orbitals the valence band emerges, bordered by the highest occupied molecular orbital (HOMO), while the π*-orbitals form the conduction band, which is bordered by the lowest unoccupied molecular orbital (LUMO). The energy gap between the HOMO and LUMO is called the bandgap *E*_g_. Since the σ-bonds preserve the linear-chain structure of the molecule, the resulting π-orbitals are free to undergo optical and electronic interactions where electric charge can be delocalised along the chain or transported to adjacent molecules.

**Fig. 4:**
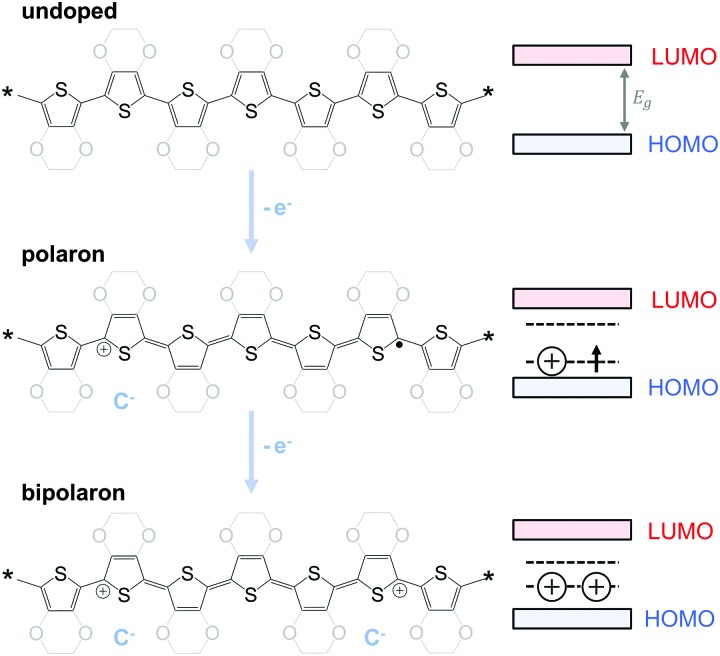
Schematic for p-doping of polythiophene and PEDOT (left) and the corresponding band structure evolution (right): electron transfer (–e^–^) to the undoped polymer (top) leads to the formation of a polaron (middle) and finally a bipolaron state (bottom); counterions (C^–^) ensure charge neutrality.

To optimise the thermoelectric performance of a semiconductor, the electrical conductivity must be increased through doping (and balanced with regard to *α* and *κ*). Doping introduces new electronic states (polarons or bipolarons) in the bandgap of the polymer (Fig. 4), which give rise to new optical transitions that can be observed as a broad infrared absorption. Doping can be achieved through either a redox or an acid–base reaction, where an electron or ion, respectively, is exchanged between the conjugated polymer and the dopant (*cf.* Section 4). In both cases, the frontier orbital energy is a contributing driving force for doping. Redox p-doping involves an electron transfer from the higher HOMO level of the conjugated polymer to the lower lying LUMO level of the dopant. For acid–base reactions, a higher HOMO level permits p-doping with weaker acids, *i.e.* the transfer of a proton (H^+^) to the conjugated polymer,^16^ while a decrease in the LUMO level of the conjugated polymer can contribute to improved n-doping efficiency through hydride transfer (H^–^).^17^

**Fig. 5:**
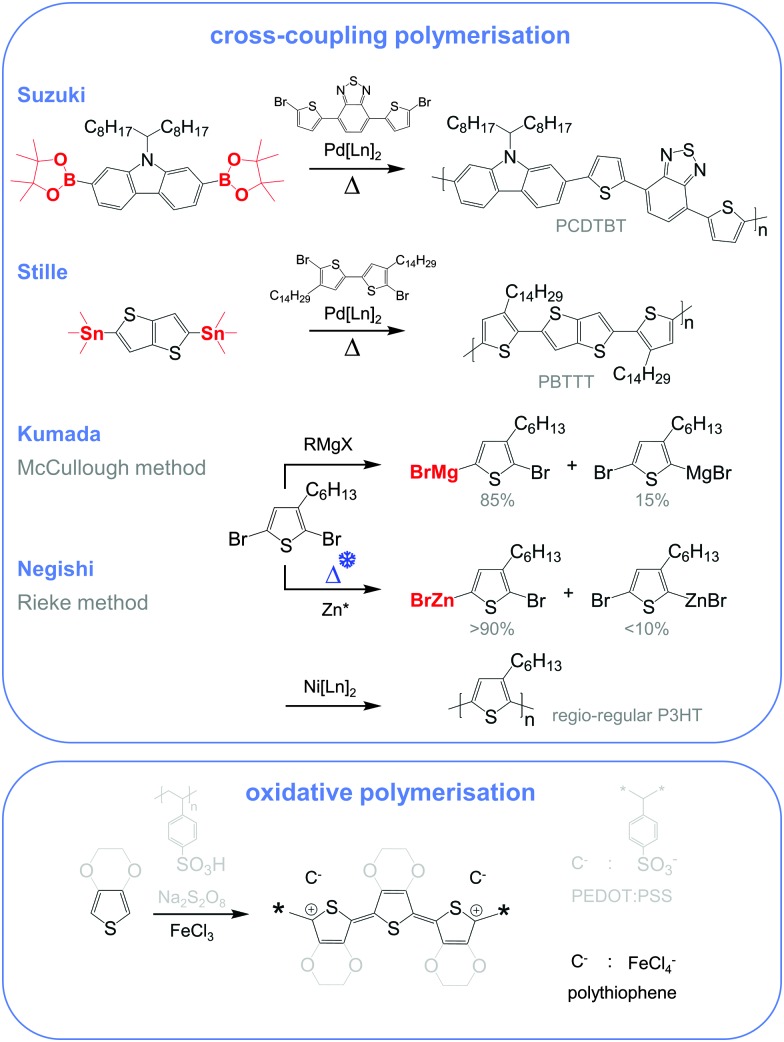
Most common reaction schemes for conjugated polymer synthesis include cross-coupling polymerisations (top), where a bifunctionalised monomer (red, boronic ester for Suzuki, organostannyl for Stille, *Grignard* for Kumada and organozinc for Negishi) is polymerised with an aryl halide (mostly an aryl bromide) by the action of a metal catalyst, and oxidative polymerisation (bottom) of 3,4-ethylenedioxythiophene (grey) and thiophene (black) yielding PEDOT:PSS and doped polythiophene.

**Fig. 6:**
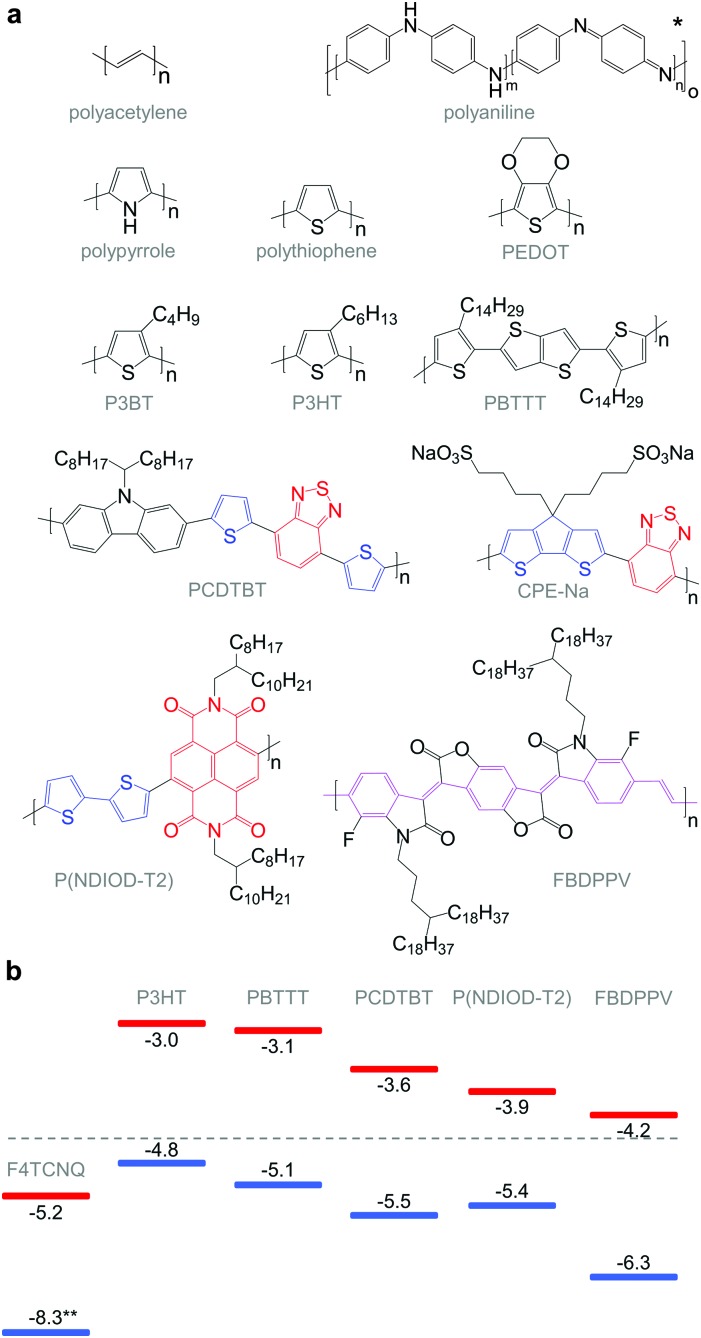
(a) Chemical structures of conjugated polymers discussed in this review. For the donor–acceptor architectures, donor and acceptor moieties are indicated in blue and red, respectively. The PPV-backbone in FBDPPV is indicated in purple; * leucoemeraldine (*m* = 1, *n* = 0), emeraldine base (*m* = *n* = 0.5), pernigraniline (*m* = 0, *n* = 1); (b) HOMO and LUMO levels of P3HT, PBTTT, PCDTBT, P(NDIOD-T2) and FBDPPV, as well as the oxidising agent F4TCNQ; ** not to scale.

Molecular engineering can be employed to adjust the HOMO and LUMO levels with regard to a particular dopant. A wide spectrum of design tools are available, such as (1) the donor–acceptor approach to narrow *E*_g_, (2) electron-withdrawing units to narrow *E*_g_, (3) fused heterocycles to increase backbone planarity and extend delocalisation, and (4) two-dimensional conjugation.^18^ An early report by Leclerc *et al.* found that the donor–acceptor approach can lead to materials that simultaneously display a high electrical conductivity and Seebeck coefficient.^10^ The authors polymerised several alkyl-substituted carbazoles through Suzuki coupling, which is a much less toxic alternative to Stille polymerisation that is usually employed because of the structural versatility and stability of stannyl-functionalised, thiophene-based monomers (Fig. 5). The carbazole–benzothiadiazole copolymer PCDTBT (Fig. 6) displayed – upon p-doping with FeCl_3_ – the highest thermoelectric performance with *σ* ∼ 160 S cm^–1^, *α* ∼ 34 μV K^–1^ and thus *α* ^2^ *σ* ∼ 19 μW m^–1^ K^–2^.

### 2.2. Melt and solution processability

Unsubstituted conjugated polymers such as polythiophene, polypyrrole, polyaniline and PEDOT are intractable, especially when subsequently doped. These materials can be either processed in the form of a powder that can be compacted to form a solid object, or deposited *in situ* as a thin film on an electrode through electrochemical polymerisation. Post-synthesis processability can be achieved through the formation of a soluble polymer:dopant complex. One example is the emeraldine base of polyaniline (Fig. 6), which can be synthesised through oxidative polymerisation of aniline that upon protonation of the imine nitrogens forms the highly conductive and air-stable emeraldine salt. Cao *et al.* have shown that acid doping with CSA or dodecylbenzenesulfonic acid (DBSA) leads to counterion induced processability as evidenced by good solubility of the resulting polymer:dopant complex in polar solvents such as *N*-methylpyrrolidone (NMP) and *m*-cresol.^19^ Another example is the extensively studied complex of PEDOT and the solubilising counterion PSS, which is formed through oxidative polymerisation of 3,4-ethylenedioxythiophene with sodium persulfate as the oxidising agent in the presence of PSS (*cf.* Section 7) (Fig. 5).^20^ The resulting PEDOT:PSS complex can be processed from water, which is an excellent choice of solvent because of its low environmental footprint.

One widely used approach to enhance the processability of conjugated polymers from their melt or (less environmentally benign) organic solvents is the decoration with alkyl side chains, with the family of poly(3-alkylthiophene)s (P3ATs) as a prominent example. Oxidative polymerisation of P3ATs (Fig. 5) results in regio-random coupling of monomers and therefore yields an amorphous polymer where regio-defects limit the delocalisation of electrons across adjacent π-orbitals. Instead, the synthesis of P3HT *via* Kumada (McCullough method) or Negishi (Rieke method) cross-coupling polymerisation (Fig. 5) allows – despite the formation of an isomeric monomer mixture – the formation of a highly regio-regular backbone that renders the polymer semi-crystalline. The melting and glass transition temperatures decrease with the alkyl side chain length from *T*_m_ > 300 °C and *T*_g_ ∼ 100 °C for polythiophene to, for instance, *T*_m_ ∼ 220–250 °C and *T*_g_ ∼ 10 °C for regio-regular poly(3-hexylthiophene) (P3HT).^21,22^ At the same time the solubility in organic solvents is dramatically enhanced, which not only benefits processing but also the synthesis of higher molecular-weight polymers. P3HT is considered to provide the best compromise between good processability and electronic properties, since an unnecessarily long insulating side chain compromises the amount of electronically active conjugated backbone. Other polythiophenes such as the more rigid thienothiophene-containing PBTTT (Fig. 6), which also features a lower side chain grafting density, are less soluble and thus require longer alkyl side chains. Due to the electron-rich nature of the thiophene repeat unit, polythiophenes such as P3HT and PBTTT display a high HOMO level, which forms an energetically well-aligned redox combination with common molecular dopants. Doping can strongly reduce the processability of a conjugated polymer due to a strong decrease in solubility and an increase in their *T*_g_ and *T*_m_ because of stiffening of the polymer backbone.

Functionalisation of side chains with acidic or basic groups results in the creation of so-called conjugated polyelectrolytes (CPE), which are attractive due to their solubility in water in combination with the ability to self-dope the polymer backbone. Self-doping is an interesting design strategy because elimination of a separate dopant considerably simplifies processing and allows doping of thick structures. One recent example was reported by Mai *et al.* who synthesised a series of cyclopenta-dithiophene-*alt*-benzothiadiazole copolymers that carried alkyl-sulfonate side chains (*cf.* Fig. 6 for example, CPE-Na).^23^ Alkyl-sulfonate side chains act as negative counterions that stabilise positive polarons on the polymer backbone. The type of counterion and side chain length were found to have a moderate influence on the thermoelectric properties of these conjugated polyelectrolytes. Sodium cations gave rise to a *σ* ∼ 0.2 S cm^–1^, which paired with a high *α* ∼ 200 μV K^–1^ led to a power factor close to 1 μW m^–1^ K^–2^. The side chain design appeared to have little effect on the thermal conductivity with values around 0.2 to 0.3 W m^–1^ K^–1^.

### 2.3. Chain length

One of the most important characteristics of a polymer is its chain length, or molecular weight, which critically influences its solubility and viscosity, and therefore its processability from solution and melt. Moreover, the mechanical and electronic properties are strongly linked to the nanostructures that tend to develop in materials with different molecular weights (*cf.* Section 3). Synthesis is typically carried out in solution, where monomers join to form a polymer with increasing chain length until either the monomers are consumed or the polymer has reached a molecular weight where it becomes insoluble. Typically, a distribution of chain lengths is obtained, which is summarised by the polydispersity index, *i.e.* the ratio of weight-average and number-average molecular weight, PDI = *M*_w_/*M*_n_. It is important to remember that reported molecular weights typically represent relative values that were measured using size-exclusion chromatography (SEC) against a calibration standard such as polystyrene, which for conjugated polymers leads to a significant overestimate (*e.g.*, a factor of two for P3HT).

## 3. Solid-state structure–property relationships of conjugated polymers

The impact of the solid-state nanostructure on the mobility of charge carriers *μ* has been extensively studied in thin-film opto-electronic devices such as diodes and FETs where an electric field is used to induce charge carriers into the semiconductor. Many materials are known to be ambipolar, *i.e.* they can conduct both electrons and holes (materials that oxidise in air tend to only display p-type behaviour). If the charge carrier concentration is low, charges become localised by traps that exist due to energetic disorder, and thus their mobility can be as low as 10^–5^ cm^2^ V^–1^ s^–1^. With increasing charge carrier concentration, once all traps are filled, hopping of charges between energetic sites can occur more freely and a much higher mobility is obtained, which represents an upper bound for the mobility in more heavily doped conjugated polymers.^4^ Many conjugated polymers are now available that reach trap-free electron and/or hole mobilities of more than 1 cm^2^ V^–1^ s^–1^, which is comparable to inorganic semiconductors such as amorphous silicon.

Based on the analysis of several polythiophenes as well as available literature data, Glaudell *et al.* have argued that the Seebeck coefficient scales with electrical conductivity according to *α* ∝ *σ* ^–1/4^ and therefore any increase in the latter tends to improve the power factor *α* ^2^ *σ*.^2^ Since *σ* scales with *μ* (eqn (3)), we here introduce the reader to some of the key structure–property relationships relevant for charge-transport in conjugated polymers. We argue that high-mobility materials in combination with appropriate processing schemes will lead to further advances in organic thermoelectrics.

### 3.1. Molecular order

**Fig. 7:**
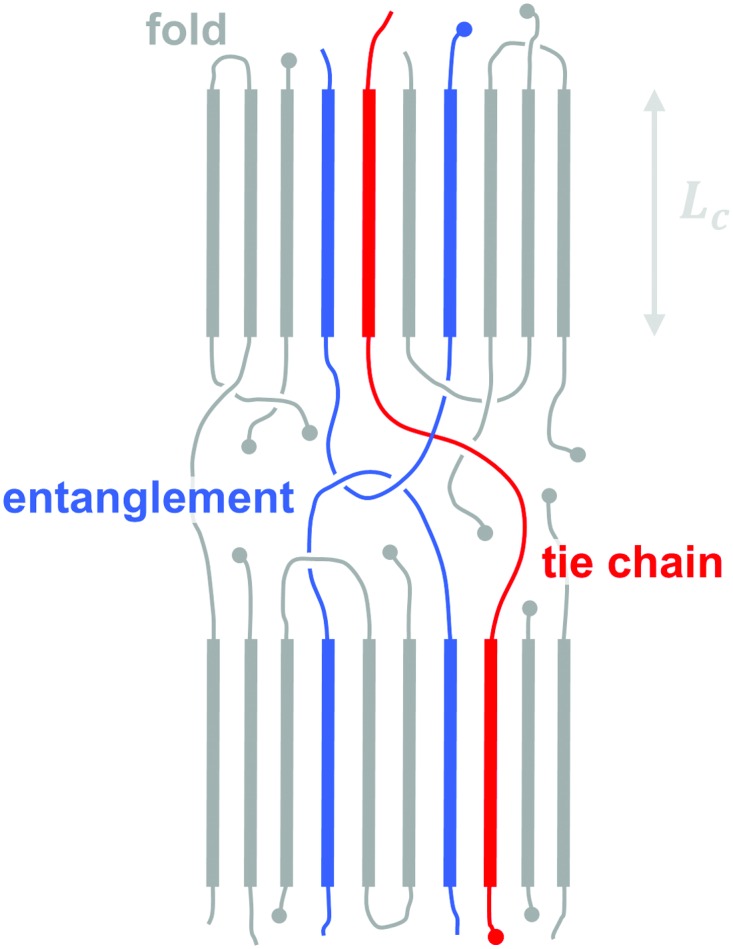
Schematic nanostructure of a semi-crystalline polymer, indicating a chain fold that re-enters the same crystallite (with lamellar thickness *L*_c_), a tie chain that bridges two adjacent crystallites, and a chain entanglement that is trapped in the amorphous region between two crystallites.

All (conjugated) polymers that feature a regular sequence of repeat units possess the ability to order. In analogy to classical polymers such as polyolefins, concepts such as crystallinity, *i.e.* the volume fraction of the crystalline phase, and lamellar thickness, *i.e.* the thickness of a crystalline domain, can be used to describe the degree of ordering and size of ordered domains (Fig. 7). Lamellae typically consist of co-facial stacks of polymer segments that form because of strong π-orbital overlap. Due to the high rigidity of many conjugated polymers ordered domains are best described by a fringed micelle-like architecture, where the same macromolecule is unlikely to re-enter the same crystallite.

Regio-regular P3HT is the most extensively studied semi-crystalline conjugated polymer. P3HT readily crystallises when processed from solution or the melt but tends to feature a fine distribution of crystallites with numerous grain boundaries that impede charge transport and typically limit the FET mobility to values around 10^–2^ cm^2^ V^–1^ s^–1^. Crossland *et al.* have shown that careful control of nucleation can lead to the growth of micrometre-sized spherulites, *i.e.* a spherical superstructure of crystallites that originate from a common nucleus. The associated reduction in the number of spherulite boundaries resulted in a higher charge carrier mobility of about 10^–1^ cm^2^ V^–1^ s^–1^.^24^

A number of conjugated polymers feature a more rigid polymer backbone than P3HT and thus can form liquid-crystalline phases above *T*_m_, where local alignment of the conjugated backbone gives rise to long-range order. A prominent example is PBTTT, which can assemble into several 100 nanometre large ordered domains on solidification from a liquid-crystalline phase. The associated decrease in the number of grain boundaries results in a significant increase in charge carrier mobility to values as high as 0.6 cm^2^ V^–1^ s^–1^.^25^

The degree of solid-state ordering of a conjugated polymer tends to strongly depend both on the molecular structure as well as on the chosen processing conditions. For instance, rapid solidification from solution or melt combined with a high glass transition temperature can force a regular polymer to adopt a less ordered nanostructure.^22^ Furthermore, a number of conjugated polymers developed *e.g.* for organic FETs and solar cells do not display pronounced long-range crystalline order when studied using standard X-ray scattering techniques, but nevertheless sustain high charge carrier mobilities facilitated by the strong tendency of conjugated backbones to π-stack.^26^ The nanostructure of these materials is characterised by pronounced short-range order involving only a few chain segments at a time.

### 3.2. Molecular weight, tie chains and entanglements

The molecular weight can have a pronounced effect on the charge carrier mobility. For example, the charge carrier mobility of P3HT first strongly increases with molecular weight but saturates at *M*_n_ > 25 kg mol^–1^.^21^ The mechanical properties provide a strong indication as to the underlying structural changes. Short-chain oligomers, which form extended-chain but disconnected crystalline domains, give rise to a brittle material. Instead, higher molecular-weight P3HT is ductile and deforms under tensile stress, which indicates an interconnected network due to the presence of (trapped) entanglements and tie chains that connect adjacent crystallites and can carry mechanical load (Fig. 7).

The impact of connectivity on electronic charge transport can be understood by considering how charges that travel through a semi-crystalline polymer encounter both ordered and disordered domains, for which Mollinger *et al.* have recently developed a concise model.^27^ Within crystallites charge transport is rapid and thus the local mobility is high. In disordered regions, however, it is necessary to distinguish between movement along the same chain and charge hopping between chains, with the intra-chain mobility being considerably faster than the inter-chain mobility. As a result, the make-up of the disordered region is of critical importance for efficient charge transport. In the case of oligomers or low molecular weight polymers, slow charge transport across the grain boundaries between disconnected crystalline domains has a detrimental effect on the overall mobility even if the degree of ordering is high. Instead, sufficiently long polymer chains are able to form tie chains that bridge adjacent crystallites (Fig. 7). Transport along tie chains permits charges to traverse the disordered region between crystallites more rapidly, which can explain the high mobility of less ordered polymer semiconductors.^27^ When considering the impact of molecular weight, it is important to take into account the whole molecular weight distribution. Even a relatively low amount of high molecular weight material may provide sufficient connectivity to improve charge carrier mobility.

Besides the positive impact on the electronic charge carrier mobility, the presence of entanglements and tie chains also leads to ductile conjugated polymers. As discussed above, higher molecular-weight P3HT permits plastic deformation accompanied by considerable chain alignment, with an elongation at break close to 300% for *M*_n_ ∼ 100 kg mol^–1^.^21^ Other conjugated polymers that offer a higher charge carrier mobility also tend to feature a more rigid backbone as compared to P3HT, leading to an above ambient *T*_g_, which must be surpassed to recover ductile behaviour.^22^

### 3.3. Texture of ordered domains

**Fig. 8:**
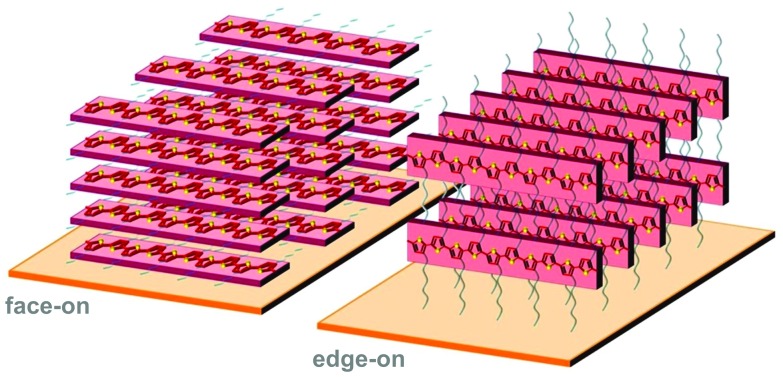
Face-on (left) and edge-on (right) orientation of a P3HT crystallite with the π-stacking direction lying out-of-plane and in-plane, respectively.

Polymer crystallites can either be randomly oriented with respect to each other or display preferential orientation, which is referred to as crystalline texture. Solution-cast thin films often feature at least some degree of texture, which is usually expressed in terms of the orientation of the conjugated backbones relative to the substrate (face-on and edge-on; Fig. 8). Intermolecular charge transport is favoured in the direction of π-stacking and, therefore, the in-plane and out-of-plane mobility tend to differ. For instance, in devices such as polymer solar cells transport of generated charges occurs in the out-of-plane direction and, therefore, a preferential face-on texture is favoured.^28^ Similarly, Palumbiny *et al.* recently observed that the in-plane electrical conductivity of PEDOT:PSS increased from 0.2 to 1200 S cm^–1^ upon treatment with ethylene glycol, which was attributed to the formation of larger PEDOT crystallites with more pronounced edge-on orientation.^29^

### 3.4. Uniaxial alignment

A number of tools exist that permit the uniaxial alignment of conjugated polymers, including thin-film techniques such as the use of liquid-crystal alignment layers, directional epitaxial growth and imprinting, as well as bulk techniques such as solid-state pressing and tensile drawing.^21,30^ Bulk processing is likely to be of particular interest in the context of organic thermoelectrics since it is possible to realise the required millimetre-thick structures.

A number of reports have explored the impact of stretch alignment on the thermoelectric performance. As early as 1990, Nogami *et al.* studied uniaxially aligned iodine-doped polyacetylene.^8^ A very high *σ* ∼ 60 000 S cm^–1^ and *α* ∼ 15 μV K^–1^ gave rise to a power factor of about 1350 μW m^–1^ K^–2^ (Table 1), which is until today one of the best results for an (albeit poorly stable) organic thermoelectric material. Mateeva *et al.* measured the *σ* and *α* of stretch-aligned polyaniline and polypyrrole and found that for the same Seebeck coefficient the electrical conductivity was two orders of magnitude higher along the direction of orientation.^31^

Thermal transport in stretch-aligned conjugated polymers has not been thoroughly investigated. High-modulus fibres of *e.g.* ultra-high molecular weight polyethylene (UHMWPE) or polyaramides, which are characterised by a very high degree of alignment, are known to display a high thermal conductivity of more than 10 W m^–1^ K^–1^ along the long axis of the fibres. Therefore, it can be anticipated that aligned conjugated polymers display a similar behaviour.

## 4. Doping of conjugated polymers

**Fig. 9:**
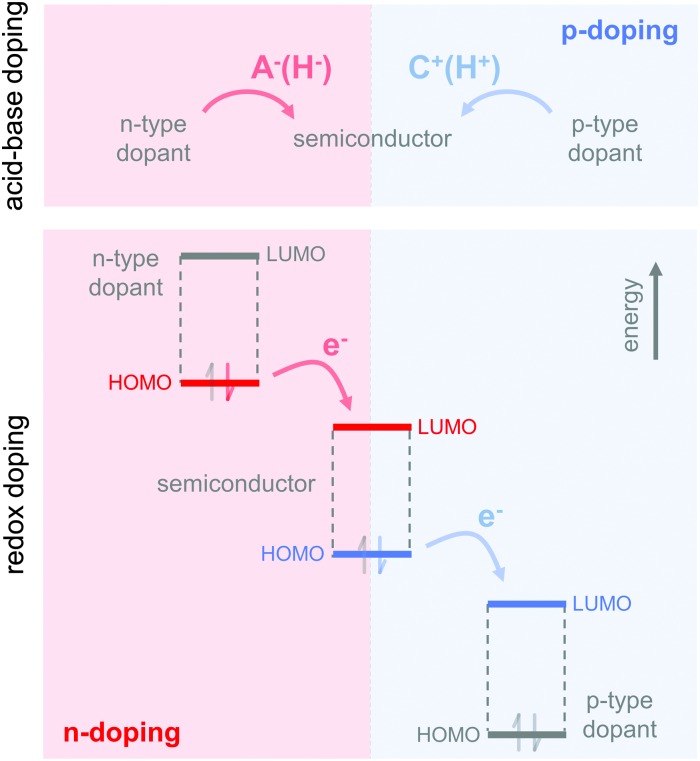
Basic principle of acid–base doping, which involves the transfer of an anion (A^–^, *e.g.*, H^–^) or cation (C^+^, *e.g.*, H^+^) to the semiconductor (top); and redox doping, which involves the transfer of an electron to the LUMO or from the HOMO of the semiconductor in the case of n- and p-doping, respectively (bottom).

**Fig. 10:**
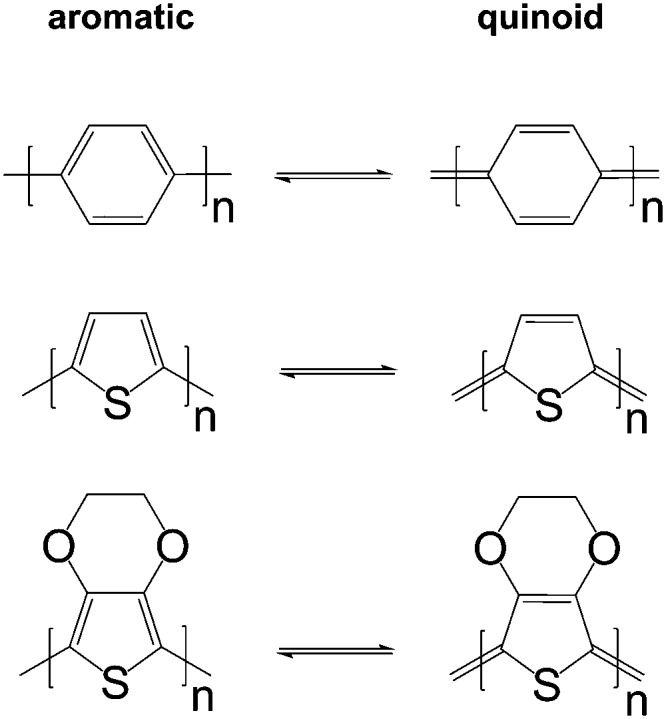
Aromatic-quinoid resonance of poly(*p*-phenylene) (top), polythiophene (centre) and PEDOT (bottom).

The charge carrier concentration can be modulated through the addition of a dopant, *i.e.* a “charge-transfer agent used to generate, by oxidation or reduction, positive or negative charges in an intrinsically conducting polymer” (IUPAC gold book definition for dopant). Doping can be achieved by two means: (1) redox doping that involves the transfer of electrons, to form a donor–acceptor charge-transfer complex or ion pair, and (2) the transfer of a cation or anion to the polymer backbone, which we refer to as acid–base doping (Fig. 9). In the latter case, charge neutrality is maintained by the presence of a counterion. Through the addition or removal of one electron, or the addition of an anion or cation, negative (radical anion) or positive (radical cation) polarons are created which, at higher doping levels, combine to form bipolarons (Fig. 4). Both polarons and bipolarons are delocalised over several repeat units. The associated change in the local structure of the polyaromatic backbone is thought to be accompanied by a more quinoidal conformation (Fig. 10). The HOMO and LUMO levels of conjugated polymers but also of filler materials such as carbon nanotubes determine the doping efficiency, *i.e.* the number of charges generated per dopant molecule (*cf.* Sections 2 and 6).^16,32,33^

The doped state can be sensitive to environmental parameters such as air and light as well as heat. Whereas encapsulation can protect from exposure to air and light, thermal gradients cannot be avoided since they are essential for the functioning of a thermoelectric device. Therefore, for practical use a high degree of thermal stability of the doped state must be ensured in order to avoid gradual loss of the thermoelectric performance of an initially optimised material.

### 4.1. Acid–base doping through ion transfer

**Fig. 11:**
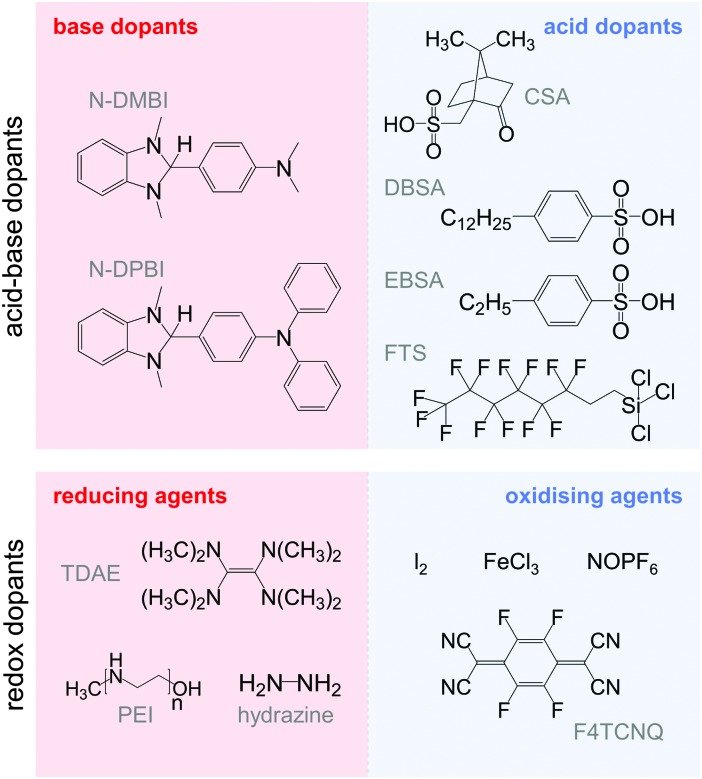
Acid–base (top) and redox dopants (bottom) discussed in this review.

The transfer of a proton (H^+^) to the backbone of a conjugated polymer is commonly referred to as acid doping and results in a p-type material (Fig. 9). A prominent example is the emeraldine base of polyaniline, which turns into the highly conductive emeraldine salt upon protonation using CSA or DBSA, with an electrical conductivity of more than 100 S cm^–1^.^19^ Other examples of polymers that can be acid-doped are polythiophenes such as P3HT and PBTTT. Various doping strategies have been successfully employed such as immersion of PBTTT in ethylbenzenesulfonic acid (EBSA) or exposure of polythiophene to the vapour of perfluorooctyltrichlorosilane (FTS) (Fig. 11). In the latter case, a protonic doping mechanism is thought to occur through hydrolysis of the chlorosilane bond, accompanied by the formation of hydrochloric acid.^34^

Benzimidazole derivatives such as N-DMBI and N-DPBI depicted in Fig. 11 are air-stable compounds that permit n-doping of organic semiconductors through hydride (H^–^) transfer in an inert atmosphere (Fig. 9). In analogy to acid doping, we refer to this process as base doping. Schlitz *et al.* demonstrated that the napthalenedicarboximide-bithiophene copolymer P(NDIOD-T2) (Fig. 6) can be base-doped with both N-DMBI and N-DPBI, which is facilitated by the low LUMO level of the polymer around –3.9 eV.^35^ Electrical conductivities of up to 0.008 S cm^–1^ paired with a high negative Seebeck coefficient of –850 μV K^–1^ were achieved giving rise to a power factor of 0.6 μW m^–1^ K^–2^, although only one out of 100 doping molecules was thought to lead to hydride transfer. Similarly, n-doping with N-DMBI of a series of electron-deficient benzodifurandione-based poly(*p*-phenylene vinylene) derivatives (BDPPV) with LUMO levels around –4.0 to –4.3 eV yielded a promising n-type behaviour.^17^ The fluorinated derivative FBDPPV (Fig. 6) displayed a power factor of up to 25 μW m^–1^ K^–2^, which is among the best results obtained for n-type materials to date (Table 1).

### 4.2. Redox doping through donor-to-acceptor electron transfer

Redox doping involves the (partial) transfer of an electron from a donor to an acceptor molecule, which leads to the formation of an ion pair (or charge-transfer complex in the case of partial transfer). Transfer of an electron from the HOMO of the conjugated polymer to the LUMO of the acceptor gives rise to p-doping, while transfer from the HOMO of a donor to the LUMO of the semiconductor leads to n-doping (Fig. 9). Effective redox doping requires a slight offset between the involved HOMO and LUMO levels. As mentioned previously, iodine doping of polyacetylene is the first reported example of strong p-type redox doping (*cf.* Section 2).^8^ Unfortunately, polyacetylene is characterised by poor environmental stability. Therefore, redox p-doping of more stable conjugated polymers such as polycarbazoles, polyfluorenes and polythiophenes with a number of oxidising agents including FeCl_3_, NOPF_6_ and F4TCNQ (Fig. 11) has been explored extensively. Yim *et al.* have studied the influence of the HOMO level on the doping strength when processing the conjugated polymer and dopant from the same solution.^32^ Among the investigated polymers, the highest conductivity of ∼10^–1^ S cm^–1^ was achieved with P3HT due to an adequate offset between the HOMO of –4.8 eV and LUMO of –5.2 eV of P3HT and F4TCNT, respectively. More recently, Glaudell *et al.* investigated solution doping of several polythiophenes with F4TCNQ.^2^ PBTTT, which features a slightly lower HOMO of –5.1 eV, gave rise to promising values of *σ* ∼ 3 S cm^–1^ and *α* ∼ 60 μV K^–1^.

### 4.3. Doping-induced structural changes

A critical aspect to consider when evaluating doping reactions of conjugated polymers is that the dopant can reach several tens of mole percent before optimal thermoelectric properties are observed. This is in contrast to doping of inorganic semiconductors, where the dopant only constitutes a trace amount. The inclusion of such large dopant fractions is typically accompanied by a concomitant change in the nanostructure of the semiconductor through intercalation of dopant molecules between polymer chains.^2^ Examples include F4TCNQ:P3HT crystals as well as PEDOT:Tos, both of which feature a different crystalline unit cell compared to the neat polymer. Evidently, doping can have a substantial impact on the micro- and nanostructure of a conjugated polymer, which will strongly alter its energetic landscape as described by the density of states. Thus, a doped organic semiconductor should be treated as a new material. Comparison with inorganic semiconductors, for which the magnitude of the Seebeck coefficient correlates with charge carrier concentration, should be done with caution. To gain a detailed understanding of relevant structure–property relationships that describe doping of conjugated polymers, it is important to combine an in-depth characterisation of the thermoelectric properties with detailed spectroscopic and structural studies, including *e.g.* X-ray diffraction and electron and/or atomic force microscopy.

### 4.4. Dopant:semiconductor processing strategies

Co-processing of conjugated polymers and a large dopant fraction from the same solution potentially suffers from gross post-deposition phase separation due to incompatibility of the two components. Conversely, if charge transfer between the polymer and dopant already occurs in solution then precipitation can occur. Both issues need to be addressed as they limit the processability of a given polymer:dopant combination and lead to suboptimal nanostructures. Several strategies can be exploited that permit the mitigation of these processability issues. Counterion-induced processability of polyaniline and otherwise intractable PEDOT has been discussed in Section 2. A related approach involves self-doping of conjugated polyelectrolytes, where the dopant is covalently bound to the side chain of the semiconductor.^23^

To gain control over structure formation, it is desirable to select processing schemes that permit the conjugated polymer to first solidify from solution or melt, followed by a separate doping step. For instance, the early work of Nogami *et al.* and Mateeva *et al.* explored the influence of uniaxial orientation on the thermoelectric parameters.^8,31^ Neat solution-cast films of polyacetylene and polyaniline were subjected to tensile drawing, subsequent to which the oriented samples were exposed to iodine vapour and acid solutions, respectively. Such treatment prior to the deformation step would have complicated tensile drawing. Instead, treatment of the final structure with dopant vapour or the dopant dissolved in an orthogonal solvent permitted to disentangle the processing and doping step. A recent example is the work by Patel *et al.* who studied the thermoelectric properties of PBTTT thin films doped through exposure to FTS vapour or immersion in an EBSA solution, reaching high values of *σ* ∼ 10^3^ S cm^–1^, *α* ∼ 33 μV K^–1^ and thus a high power factor of 110 μW m^–1^ K^–2^ for FTS-doping.^34^ The question arises to which extent the dopant would be able to penetrate bulk samples, which are likely to be required for optimally designed thermoelectric modules. We conclude that strategies, which permit effective doping independent of the sample dimensions, must be developed in order to enhance the scope of organic thermoelectric plastics.

## 5. Polymer semiconductor:insulator blends

A practical organic thermoelectric material should, besides a high figure or merit, also offer good processability as well as mechanical robustness. Solution and melt processing techniques, such as screen and 3D printing, that permit the realization of at least 100 micrometre thick patterns of optimally designed thermoelectric generators (*cf.* Section 8) require viscous inks and pastes. In addition, the fabrication of flexible and/or stretchable devices will be eased if elastic conductors are available that can reversibly accommodate the large strains, which arise upon mechanical deformation. However, the low degree of polymerisation of many conjugated polymers severely limits their viscoelastic properties. Doping, which is essential to tune the charge-carrier concentration (*cf.* Section 4), tends to further increase their brittleness.^36^

Blending of conjugated polymers (or small-molecular organic semiconductors such as fullerenes and perylene diimides) with insulating polymers is a powerful tool that permits the adjustment of the rheological and mechanical properties. A number of studies have explored this concept for thin-film organic opto-electronic devices ranging from FETs to polymer solar cells.^37^ The addition of a low amount of a high molecular-weight binder can be used to increase the viscosity of a processing solution. Instead, a higher amount of insulating polymer can act as a matrix that provides elasticity or ductility as well as encapsulation, which enhances environmental stability. Importantly, judicious selection of appropriate processing routes permits to maintain the electronic performance of the neat semiconductor. For instance, Kumar *et al.* investigated the bulk charge transport in micrometre-thick films of blends comprising regio-regular P3HT and high-density polyethylene (HDPE), and found that the charge carrier mobility remained unaffected upon addition of as much as 90 wt% of the insulator.^38^ A study by Ferenczi *et al.* demonstrated that the addition of 40 wt% HDPE to a P3HT:fullerene bulk-heterojunction blend can result in considerably improved mechanical robustness and permits the fabrication of thicker active layers that display the same photovoltaic performance as the neat donor:acceptor mixture.^37^ It should be noted that electrical percolation can occur at much lower fractions of the conjugated polymer. An early example by Cao *et al.* established that electrical percolation at a polyaniline:DBSA fraction of less than 2% can be achieved in a variety of insulating matrices, which could be composed of apolar polymers such as polyethylene, polypropylene and polystyrene (PS) or more polar ones such as poly(vinyl chloride) (PVC), poly(methyl methacrylate) (PMMA), polycarbonate, polyamides and PVA.^19^

The use of insulating polymers for the preparation of organic thermoelectric materials has been largely restricted to enhancing the processability of otherwise intractable compounds and fillers. Examples include processing of *e.g.* the electrically conducting salt tetrathiafulvalene:tetracyanoquinodimethane (TTF:TCNQ) through blending with PVC^12^ and carbon nanotubes in a PVA^11^ or polyvinylidene difluoride (PVDF) matrix.^39^ Lu *et al.* have carried out a comprehensive study on the thermoelectric properties of poly(3-butylthiophene):PS (P3BT:PS) blends.^40^ A carefully selected processing protocol resulted in the growth of oxygen-doped P3BT nanofibers from solution that formed an interpenetrating network embedded in a PS matrix. Interestingly, the electric conductivity and power factor improved compared to the neat semiconductor giving rise to a *ZT* ∼ 10^–4^ for an optimal PS content of 60 wt%. Evidently, the use of appropriately selected matrix polymers not only expands the processing window of conjugated polymers but also permits the optimisation of their thermoelectric performance. Additional benefits may arise if polar matrix polymers are employed, which can display considerable ionic conductivity (*cf.* hydrated PSS in Section 7).^15^ The associated ionic contribution to the overall Seebeck coefficient may allow to further increase the figure of merit of thermoelectric plastics.

## 6. Polymer nanocomposites

One strategy towards high-performance thermoelectric plastics is the preparation of nanocomposites that comprise filler materials such as carbon nanotubes, graphene and inorganic nanoparticles, dispersed in a polymer matrix. A variety of polymer:filler combinations have been explored to date. We refer the reader to a recent review by McGrail *et al.* for a more complete overview.^41^ Here, we focus on two particular promising classes based on carbon nanotubes and tellurium nanowires in order to illustrate the potential of thermoelectric nanocomposites.

### 6.1. Carbon nanotubes

Carbon nanotubes have received tremendous attention as filler materials for polymer nanocomposites because of their high mechanical strength as well as high thermal and electrical conductivities (intrinsic values: *σ* > 10^4^ S cm^–1^ and *κ* > 10^3^ W m^–1^ K^–1^). There are two principal forms of carbon nanotubes: single-walled carbon nanotubes (SWNTs), which consist of a single sheet of graphene rolled up at a discrete chiral angle into a cylinder with a typical diameter of 1.2 to 1.4 nm, and multi-walled carbon nanotubes (MWNTs), which consist of several single tubes nested inside each other. Whereas SWNTs can be either semiconducting or metallic in character, depending on the chirality, MWNTs tend to be metallic. Several disparate synthesis methods exist, which lead to different chiralities and lengths. SWNTs are usually obtained as a mixture of semiconducting and metallic tubes, which are challenging to separate economically. The length can reach several micrometres. Carbon nanotubes display a strong tendency to aggregate into bundles, which necessitates the use of surfactants to achieve good dispersion in organic solvents or a polymer matrix.

Carbon nanotubes are of interest for thermoelectric applications because of their very high intrinsic electrical conductivity. Due to the high aspect ratio of carbon nanotubes, the onset of electrical conductivity can occur at a very low fraction of the filler. Although ambipolar behaviour can be observed in vacuum, oxygen-doping leads to unipolar p-type carbon nanotubes when exposed to air. Treatment with various oxidising or reducing agents allows the tuning of the p- or n-type behaviour, respectively. Nonoguchi *et al.* screened a library of dopants with HOMO levels ranging from –4 to –7 eV.^33^ Seebeck coefficients ranging from +90 to –80 μV K^–1^ were measured for doped SWNTs, with air-stable n-type behaviour occurring for dopant HOMO levels in the range of –4.4 to –5.6 eV. Air-stable n-type behaviour can also be achieved by incorporating nitrogen into carbon nanotubes during synthesis (Table 1).^42^

The first thermoelectric carbon nanotube based composites were reported by Yu *et al.* who used an insulating matrix based on PVA.^11^ A filler content of 20 wt% gave rise to a high *σ* ∼ 48 S cm^–1^ and *α* ∼ 40 μV K^–1^ but low *κ* ∼ 0.34 W m^–1^ K^–1^, resulting in a promising *α* ^2^ *σ* ∼ 8 μW m^–1^ K^–2^ and *ZT* > 10^–3^ at room temperature. The insulating matrix surrounding the carbon nanotubes was identified as one factor limiting the charge transport across the polymer:filler interface. The electrical conductivity can be significantly improved by using a conjugated polymer matrix. For instance, the use of a mixture of PEDOT:PSS and PVA enabled Yu *et al.* to prepare nanocomposites that displayed a much higher *σ* ∼ 10^3^ S cm^–1^ at a filler content of 60 wt%, which combined with an *α* ∼ 40 μV K^–1^ resulted in a power factor as high as 160 μW m^–1^ K^–2^ (Table 1).^14^

**Fig. 12:**
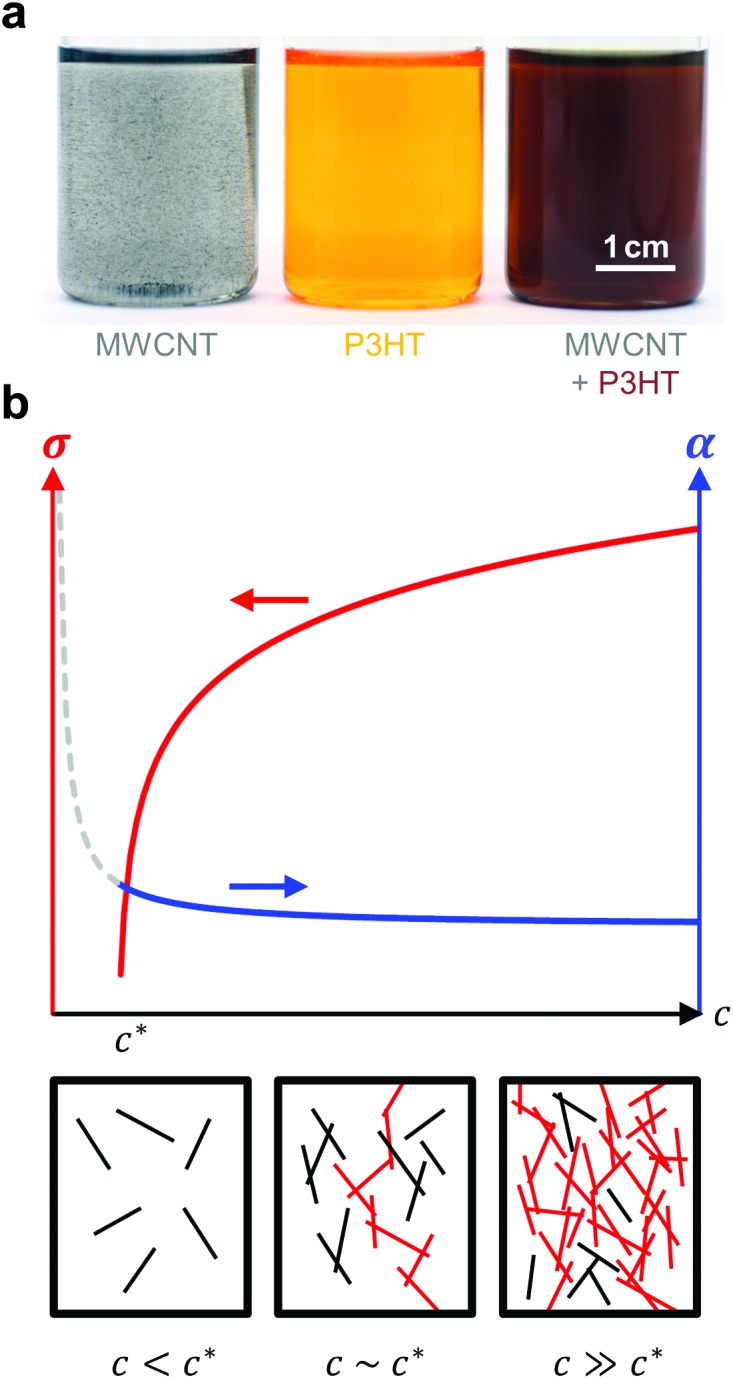
(a) Dispersion of MWNTs (left), solution of P3HT (centre), and MWNT and P3HT (right, the colour change indicates nucleation of P3HT by MWNTs); image reproduced with permission from ref. 36, published by the Royal Society of Chemistry; (b) evolution of the electrical conductivity *σ* and Seebeck coefficient *α* with concentration *c* of an anisotropic conductive filler in a less conductive polymer matrix (top); filler particles form continuous conducting pathways (red) above the percolation threshold c* (bottom).

Bounioux *et al.* measured the thermoelectric properties of P3HT:SWNT and P3HT:MWNT nanocomposites for a broad range of compositions.^36^ Both types of carbon nanotubes were found to nucleate crystallisation of P3HT, which enabled the use of the conjugated polymer as both a surfactant and a matrix (Fig. 12). Nanocomposites displayed a clear percolation threshold, *i.e.* a filler fraction above which the electrical conductivity increased by several orders of magnitude from initially 10^–5^ S cm^–1^ for neat (oxidised) P3HT to more than 10^2^ and 10^1^ S cm^–1^ in the case of SWNT and MWNT-rich composites, respectively. P3HT:SWNT nanocomposites displayed a much lower percolation threshold of 0.2 wt% (compared to 4 wt% for P3HT:MWNT), which could be rationalised with the higher aspect ratio of SWNTs. Neat P3HT featured a Seebeck coefficient of about 900 μV K^–1^, which rapidly decreased upon addition of carbon nanotubes. Above the percolation threshold, the Seebeck coefficient resembled the value measured for the neat filler, *i.e.* 12 and 32 μV K^–1^ in the case of MWNTs and SWNTs, respectively. Charge carriers predominantly travel through the more electrically conducting phase, *i.e.* the carbon nanotubes, and thus the Seebeck coefficient of the filler material is measured (Fig. 12). This behaviour can be described by a composite model that comprises two parallel conducting channels: (1) the polymer matrix, and (2) the carbon nanotube network joined by polymer barriers.

Despite the very high intrinsic thermal conductivity *κ* > 10^3^ W m^–1^ K^–1^ of single carbon nanotubes, several studies have reported a surprisingly low bulk thermal conductivity not significantly higher than that of the polymer matrix even for a high filler content, which can be rationalised with phonon scattering at the polymer/carbon nanotube interface.^11,14,36,42^ For instance, Dörling *et al.* measured a bulk thermal conductivity of 0.55 W m^–1^ K^–1^ for one millimetre-thick pellets of a P3HT:MWNT nanocomposite with a filler content of 80 wt%, which was only slightly higher than the value of 0.29 W m^–1^ K^–1^ measured for neat P3HT.^42^ However, care should be taken when evaluating the thermal conductivity of thin samples since the high aspect ratio of carbon nanotubes is likely to lead to in-plane alignment and thus anisotropic electrical and thermal conductivities.

### 6.2. Tellurium nanowires

See *et al.* have explored the use of tellurium nanowires as an inorganic filler material.^13^ The *in situ* growth of nanowires was carried out in the presence of PEDOT:PSS, which resulted in stable aqueous dispersions that could be used to drop-cast and spin-coat thin films with a tellurium content of about 85%. Nanowires were found to be coated by PEDOT:PSS, which enhanced their resistance towards oxidation in air and improved electrical contact. An in-plane *σ* ∼ 19 S cm^–1^ paired with a *α* ∼ 163 μV K^–1^ gave rise to a power factor of 71 μW m^–1^ K^–2^ (Table 1), which was found to be higher than values obtained for both neat PEDOT:PSS and tellurium nanowires. In a follow-up work, it was confirmed that the electrical conductivity peaks at a tellurium nanowire weight fraction of about 85 wt%. Instead, the Seebeck coefficient was found to increase monotonically with tellurium nanowire content. This behaviour could be described using a three-component series model consisting of (1) the polymer matrix, (2) the tellurium nanowire filler, and (3) an interfacial layer.^43^ Carrier transport was found to occur predominantly within this PEDOT:PSS interfacial layer. The authors proposed that the PEDOT:PSS coating displays a higher electrical conductivity because of a templating effect that impacts the nanostructure of the polymer complex, or energy filtering due to scattering of low energy charge carriers.

Nanocomposites of PEDOT:PSS and tellurium nanowires displayed a low out-of-plane thermal conductivity of not more than 0.3 W m^–1^ K^–1^, which is somewhat lower than the bulk thermal conductivity of tellurium of 2 W m^–1^ K^–1^.^13^ The high interfacial area between tellurium nanowires and PEDOT:PSS was thought to lead to phonon scattering, which reduces the thermal conductivity. Overall, a figure of merit *ZT* ∼ 0.1 was deduced (Table 1).

## 7. The curious case of poly(3,4-ethylenedioxythiophene)

**Fig. 13:**
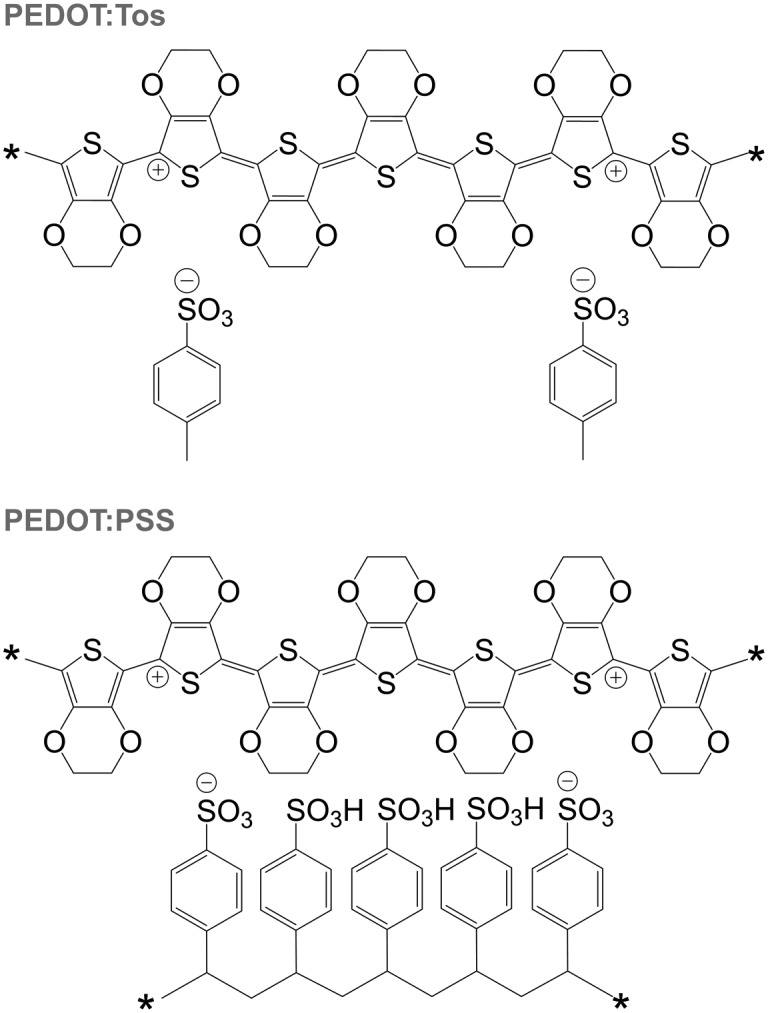
Chemical structures of PEDOT:Tos (top) and PEDOT:PSS (bottom); either chain carries one bipolaron.

PEDOT is commonly synthesised according to two principal methods *i.e.* oxidative polymerisation and electrochemical polymerisation. In the case of both methods, a conducting material is obtained since the oxidant has p-doped PEDOT during synthesis. It should be noted that the resulting conjugated oligomer PEDOT is typically of very low molecular weight and consists of 6 to 18 monomers.^20^ In the case of all oxidised forms of PEDOT, charge-balancing counterions such as Tos and PSS are present (Fig. 13). Some of the best p-type organic thermoelectric materials with a *ZT* ≥ 0.1 at room temperature are based on either PEDOT:Tos or PEDOT:PSS (Table 1),^12,44,45^ and thus PEDOT-based materials deserve a closer inspection.

According to our classification, PEDOT-based materials cover all four corners of the tetrahedron of organic thermoelectrics: (1) PEDOT is an organic semiconductor, that is (2) doped during polymerisation, and (3) can be processed together with the non-conjugated polymer PSS (Fig. 2). The fourth corner has been explored by *e.g.* See *et al.* and Yu *et al.* who used PEDOT:PSS as a conducting matrix for tellurium nanowires and carbon nanotubes, respectively (*cf.* Section 6).^13,14^

### 7.1. Optimal thermoelectric performance through reduction of PEDOT:Tos

*In situ* polymerised films of PEDOT:Tos are an excellent platform for studying the impact of the oxidation level on the thermoelectric properties. Similar to other polymer:dopant complexes, changes in crystal structure (upon introduction of the dopant or exchange of counter ions), texture as well as chain conformation (*e.g.*, aromatic to quinoid, *cf.* Fig. 10) can occur. Thus, any correlation of thermoelectric properties with the oxidation level should be accompanied by a detailed structural investigation.

In their seminal work, Bubnova *et al.* decreased the oxidation level of ∼200 nm thin PEDOT:Tos films from ∼36 to 15% through exposure to vapour of the strong reducing agent tetrakis(dimethylamino)ethylene (TDAE).^12^ A gradual decrease in electrical conductivity from *σ* > 10^2^ to <10^–4^ S cm^–1^ was accompanied by an increase in the Seebeck coefficient from *α* ∼ 40 to 780 μV K^–1^. An optimal oxidation level of 22%, at which PEDOT:Tos is air-stable, resulted in a high power factor of *α* ^2^ *σ* ∼ 324 μW m^–1^ K^–2^ at room temperature and a *ZT* ∼ 0.25 (Table 1). The high Seebeck coefficient could be rationalised with the semi-metallic properties of PEDOT:Tos, facilitated by the bipolaronic nature of charge carriers.^46^ To accurately estimate the figure of merit a careful determination of the thermal conductivity is required. Weathers *et al.* have carried out a series of in-plane measurements on free-standing PEDOT:Tos films and found that *κ*_∥_ linearly increases with *σ*_∥_ to about 1 and 1.5 W m^–1^ K^–1^ for 200 and 500 S cm^–1^, respectively.^47^ The observed increase was found to exceed the trend predicted by the Wiedemann–Franz law and the Sommerfeld value (*cf.*eqn (4) and (5)).

### 7.2. Interplay of nanostructure and thermoelectric properties of PEDOT:PSS

PEDOT:PSS is an environmentally stable material that can be processed as an aqueous dispersion. The electrical conductivity can reach values of more than 10^3^ S cm^–1^, which makes PEDOT:PSS interesting for a wide range of applications from antistatic films and transparent electrodes to conductive textile coatings and organic thermoelectrics.^20^ The rheological properties of PEDOT:PSS dispersions as well as the electrical properties of the solidified material can be adjusted by varying the solid content, PEDOT:PSS ratio (which typically ranges from 1 : 2.5 to 1 : 20), the size and distribution of gel particles and the use of additives such as surfactants or binder polymers. Stabilisers can be used to further enhance its durability.

**Fig. 14:**
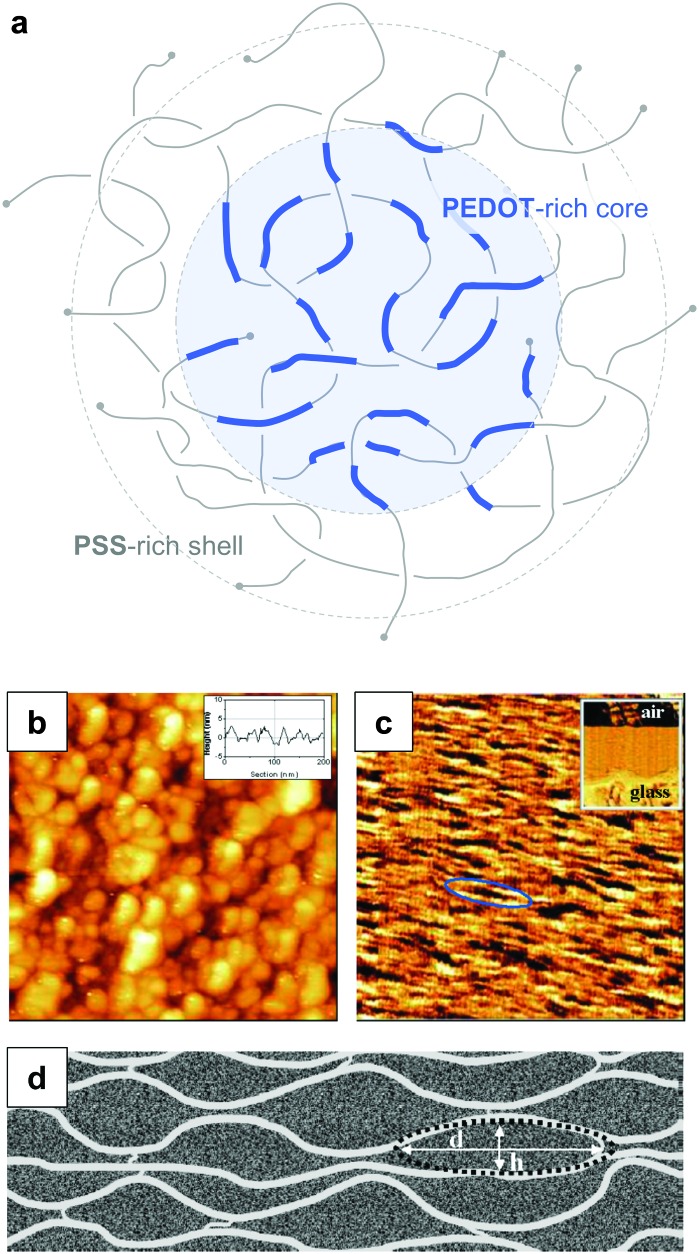
Nanostructure of PEDOT:PSS. (a) Core–shell structure of a PEDOT:PSS complex in an aqueous dispersion; (b) scanning tunnelling microscopy image of a PEDOT:PSS film surface (the inset shows a line section); (c) cross-sectional atomic force microscopy image of a cleaved PEDOT:PSS film with the glass substrate on the bottom side of the image as shown by the inset (the ellipse highlights a pancake-like particle); (d) schematic of PEDOT-rich clusters (*d* = 20 to 25 nm, *h* = 5 to 6 nm) separated by PSS lamellae; images reproduced with permission from ref. 48, published by Wiley.

PEDOT:PSS is typically processed in the form of aqueous dispersions, which are commercially available. Gel particles with a diameter of several tens of nanometres consist of a PEDOT-rich core that is surrounded by a PSS-rich outer shell (Fig. 14a).^20^ Water removal results in a solid-state nanostructure that resembles this core–shell structure. For instance, Nardes *et al.* studied thin films prepared from formulations that contained a 1 : 6 ratio of PEDOT:PSS and observed pancake-like PEDOT-rich domains that were separated by lamellae of PSS (Fig. 14b–d).^48^

The addition of polar solvents such as dimethyl sulfoxide (DMSO) and ethylene glycol to the aqueous PEDOT:PSS dispersion can have a pronounced impact on the solid-state nanostructure. Treatment of solidified films with the same solvents as well as methanol and various acids including formic and sulfuric acid can be used to modify the nanostructure. These processing agents are sometimes referred to as secondary dopants. In contrast to primary dopants, they do not affect the charge carrier concentration but the nanostructure (and hence charge carrier mobility), which persists even upon complete removal of the secondary dopants. Treatment of PEDOT:PSS thin films with secondary dopants can have a pronounced effect on the thermoelectric properties.^44,45^ Although the precise mechanism(s) that lead to structural changes are still a topic of intense study, several recurring themes can be identified, including (1) charge screening that inhibits Columbic interactions between PEDOT and PSS, (2) linearisation of PEDOT chains due to changes in chain conformation (*e.g.*, aromatic to quinoid, *cf.* Fig. 10), (3) enhanced degree of local ordering of PEDOT chains, (4) changes in the texture of PEDOT crystallites, and (5) changes in the PEDOT:PSS ratio through partial removal of PSS.

Similar to PEDOT:Tos, the oxidation level of PEDOT in PEDOT:PSS can be further modulated through the use of reducing agents such as TDAE, hydrazine and polyethylenimine (PEI) (Fig. 11). Another approach to reversibly tune the oxidation level is electrochemical oxidation/reduction. Bubnova *et al.* used PEDOT:PSS based electrochemical transistors, which permit the oxidation/reduction of the channel material by applying a negative/positive gate voltage.^49^ An increase in gate voltage to +1.4 V led to a decrease in electrical conductivity from more than 250 to 0.3 S cm^–1^, which was accompanied by an increase in the Seebeck coefficient to 400 μV K^–1^. At an intermediate gate voltage of +0.8 V an optimal compromise between *σ* and *α* was obtained, which resulted in the highest measured power factor of 24 μW m^–1^ K^–2^.

PEDOT:PSS thin films tend to be highly anisotropic (*cf.* Fig. 14b). Post-treated films often feature fibrillar domains, which likely give rise to preferential in-plane alignment. Therefore, pronounced differences between the in- and out-of-plane electrical and thermal conductivity can arise. For instance, Nardes *et al.* found that the electrical conductivity was 500 times higher in-plane.^48^ Wei *et al.* carried out a detailed study comparing the anisotropy of all three thermoelectric parameters between 20 to 150 °C.^50^ The Seebeck coefficient of 100 μm thick films appeared to be largely isotropic with *α*_∥_ ∼ 17 μV K^–1^ and *α*_⊥_ ∼ 15 S μV K^–1^ close to room temperature. In contrast, both the electrical and thermal conductivity were found to be higher in-plane with *σ*_∥_ ∼ 820 S cm^–1^ and *κ*_∥_ ∼ 0.84 W m^–1^ K^–1^ at room temperature, as compared to out-of-plane values of *σ*_⊥_ ∼ 36 S cm^–1^ and *κ*_⊥_ ∼ 0.15 W m^–1^ K^–1^. Liu *et al.* obtained similar results for the thermal conductivity.^51^ Whereas *κ*_⊥_ ∼ 0.3 W m^–1^ K^–1^ was independent of the electrical conductivity, *κ*_∥_ increased as predicted by the Wiedemann–Franz law to a value of about 1 W m^–1^ K^–1^ for *σ*_∥_ ∼ 500 S cm^–1^. The highest reported power factors for PEDOT:PSS occur at an electrical conductivity of about 10^3^ S cm^–1^, where the electronic contribution to the thermal conductivity must be taken into account (*cf.*eqn (4)).

### 7.3. Added value: mechanical robustness and ionic transport

Besides enhanced processability, PSS can add mechanical robustness to otherwise brittle PEDOT oligomers. For instance, Mengistie *et al.* prepared 100 μm thick bendable sheets that after treatment with formic acid displayed a low in-plane *α*_∥_ ∼ 21 μV K^–1^ but high *σ*_∥_ ∼ 1900 S cm^–1^, which yielded a high power factor of ∼81 μW m^–1^ K^–2^.^45^ Importantly, the brittleness of PEDOT:PSS depends on the humidity, with increased ductility at higher water uptake. Moreover, water uptake can have a strong influence on the mobility of ions in the PSS matrix. Cations are thought to diffuse through the PSS matrix along the experienced temperature gradient (Soret effect), leading to an ionic contribution to the total Seebeck coefficient. For instance, Wang *et al.* observed for PEDOT:PSS an increase in *α* above a relative humidity of 40%, with values ranging from 10 μV K^–1^ under low humidity conditions to 160 μV K^–1^ at 80% humidity.^15^ In contrast, less hygroscopic PEDOT:Tos displayed a humidity-independent Seebeck coefficient. Evidently, PEDOT-based materials hold considerable scope for future development, both in terms of mechanical robustness and further improvement of their thermoelectric properties.

## 8. Conclusions and perspectives

The number of components that are required to create promising thermoelectric plastics constitutes a formidable challenge to the materials engineer. Other branches of organic electronics such as organic photovoltaics, which mostly deals with bi-component blends, are only now starting to build a sound understanding of relevant structure–property relationships. Although those insights provide an excellent starting point for the development of organic thermoelectrics, much work is to be done to unlock their full potential.

**Fig. 15:**
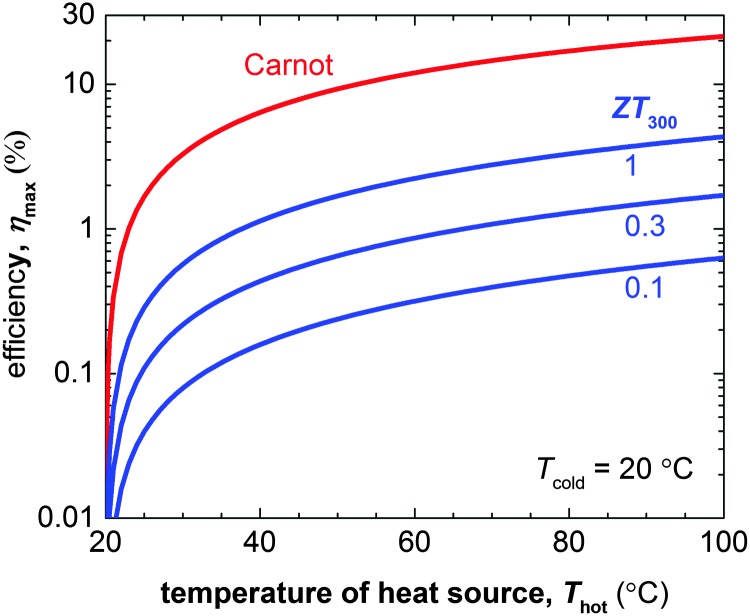
The Carnot efficiency (red) and maximum efficiency *η*_max_ of a thermoelectric leg (blue) with increasing heat source temperature *T*_hot_ (the heat sink is kept at *T*_cold_ = 20 °C). Eqn (8) was used to calculate *η*_max_ for materials with a figure of merit at 300 K of *ZT*_300_ = 0.1, 0.5 and 1.

The figure of merit of p- and in particular n-type materials is certainly one factor that needs attention. According to eqn (8) the maximum power conversion efficiency increases with the figure of merit (Fig. 15), which warrants continued efforts in optimising the thermoelectric parameters *σ*, *α* and *κ*. We argue that further significant improvements are most likely achieved if relevant structure–property relationships of *e.g.* polymer:dopant systems are studied to a greater extent. This requires a synthetic effort to systematically modify the structure of conjugated polymers as well as dopants. A better understanding of the influence of solubilising side chains, polarity, polymer molecular weight, chain conformation and energy levels will allow us to address aspects such as the poor miscibility and low doping efficiency of many molecular dopants (*cf.* Sections 2 and 4). A similar approach will permit to enhance the toolbox by which interactions between conjugated polymers and nanofillers can be tuned (*cf.* Section 6).

Furthermore, sound processing routines that permit the fabrication of samples with well controlled nanostructures are required in order to elucidate the impact of *e.g.* crystallinity and texture, entanglements and tie chains (*cf.* Section 3). Uniaxially aligned samples fabricated through *e.g.* tensile drawing, epitaxial solidification or solid-state pressing will permit the elucidation of the impact of anisotropy on charge and heat transport, which may open up avenues to decouple the mutual dependency as described by the Wiedemann–Franz law. With regard to doping, a strong limitation is the inability to separate the solidification and doping steps in many conjugated polymer systems. The development of processing schemes that permit the creation of a well-defined solid-state nanostructure, followed by controlled doping will permit the exploration of the relative impact of processing-related and intrinsic parameters. In addition, significant efforts are required with regard to the environmental stability of doped conjugated polymers. In particular, the poor air stability of doped n-type conductors will need to be addressed.

**Fig. 16:**
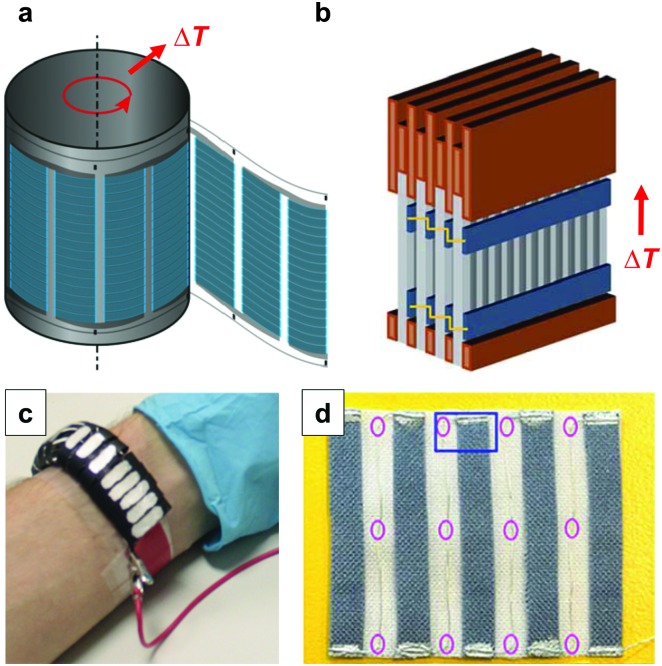
Possible designs of printed thermoelectric modules: (a) flexo-printed PEDOT:PSS based arrays on PET foil are rolled up into a cylinder; (b) screen-printed PEDOT:PSS based arrays on paper are sandwiched between copper plates and stacked into a module; (c) an array of n- and p-type legs is defined by selectively irradiating a carbon nanotube:P3HT composite with UV-light, followed by cutting and folding into a wearable toroidal device; (d) a textile-based thermoelectric generator of screen-printed PEDOT:PSS legs on a polyester fabric that are connected with conducting silver paste and silver plated copper wires as the second leg; images reproduced with permission from ref. 1, 5, 6 and 42, published by Wiley, the Royal Society of Chemistry, Wiley and the Nature Publishing Group, respectively.

Large-area printing and coating can be employed for the preparation of thermoelectric generators. Søndergaard *et al.* demonstrated roll-to-roll printing of several complete modules on poly(ethylene terephthalate) (PET) foil, which contained up to 7000 thermoelectric elements per square metre.^1^ Each element consisted of a screen-printed p-type PEDOT:PSS leg that was paired with a second flexo-printed leg of a regular conductor based on silver ink. The authors proposed that such foils can be rolled up with a heat source placed inside the resulting cylinder (Fig. 16a). Wei *et al.* used similar screen-printed architectures to build modules composed of stacks of films so that the temperature gradient can be applied in-plane (Fig. 16b).^5^ This approach allows to circumvent the mismatch between the insufficient thickness of printed films and the thick geometries that are required for optimally designed thermoelectric generators. The development of novel patterning techniques may permit to simplify the complexity of printing p- and then n-type legs. For instance, Dörling *et al.* demonstrated that selective UV irradiation of a carbon nanotube:P3HT solution can be used to switch the type of majority carrier from p- to n-type, which could be exploited to deposit both types of legs from the same solution.^42^ This patterning technique was then used for the fabrication of a wearable toroidal device (Fig. 16c). It should also be feasible to use the same ambipolar material, doped with suitable p- and n-dopants, for both types of legs.

Another interesting concept is the integration with wearable electronics. Du *et al.* demonstrated a textile-based thermoelectric generator that consisted of screen-printed PEDOT:PSS legs on a polyester fabric (Fig. 16d).^6^ Hewitt *et al.* reported an alternative in-plane architecture in the form of a felt of alternating layers of n- and p-type MWNT:PVDF composites.^39^ Such devices, which could for instance harvest small heat sources such as body heat, should not be expected to power *e.g.* mobile phones but components of tomorrow's *Internet of Things* including sensors, LEDs and RFID tags that do not consume more than a few microwatts of power. One possibility to increase the available power would be to combine thermoelectric generators with supercapacitors that charge up and then provide short bursts of power to an otherwise idle sensor. Body heat powered devices are ultimately not only limited by the efficiency of a thermoelectric generator (*η*_max_ < 1% for a skin to air Δ*T* ∼ 10 to 20 °C, *cf.* Fig. 15) but also the level of discomfort that the wearer is willing to accept due to simultaneous cooling.

Alternative device architectures should be considered that truly harness the advantages of polymer-based materials. The dimensions of an optimally designed thermoelectric generator depend on the particular application at hand. Thermal contact resistance between the thermoelectric generator and heat sink as well as the source will reduce the temperature difference that a thermoelectric leg experiences. Hence, for some applications such as wearable thermoelectric generators that exploit the temperature difference between skin and ambient air, millimetre-long legs will represent the most optimal design. The increasing availability of conjugated polymers will open up the possibility to explore textile manufacturing routines or bulk processing techniques such as most notably 3D printing, which would readily allow the fabrication of such geometries.

We conclude that thermoelectric plastics hold significant potential. The development of new materials should –besides further gains in terms of the figure of merit– focus on aspects such as availability, processability, long term stability and sustainability in order to enable the transition to a truly viable technology.
